# Chlorophyll-Amended Organoclays for the Detoxification of Ochratoxin A

**DOI:** 10.3390/toxins16110479

**Published:** 2024-11-06

**Authors:** Johnson O. Oladele, Meichen Wang, Xenophon Xenophontos, Kendall Lilly, Phanourios Tamamis, Timothy D. Phillips

**Affiliations:** 1 Interdisciplinary Faculty of Toxicology, Texas A&M University, College Station, TX 77843, USA; oladelejohn2007@tamu.edu (J.O.O.); meichenwang@umass.edu (M.W.); 2 Department of Veterinary Physiology and Pharmacology, College of Veterinary Medicine & Biomedical Sciences, Texas A&M University, College Station, TX 77843, USA; 3 Artie McFerrin Department of Chemical Engineering, College of Engineering, Texas A&M University, College Station, TX 77843, USA; xxenop01@tamu.edu (X.X.); tamamis@tamu.edu (P.T.); 4 Department of Materials Science and Engineering, College of Engineering, Texas A&M University, College Station, TX 77843, USA

**Keywords:** mycotoxin, ochratoxin, enterosorption, food contamination, thermodynamics, kinetics, isotherms, molecular dynamics

## Abstract

Climate change has been associated with outbreaks of mycotoxicosis following periods of drought, enhanced fungal growth, and increased exposure to mycotoxins. For detoxification, the inclusion of clay-based materials in food and drinking water has resulted in a very promising strategy to reduce mycotoxin exposure. In this strategy, mycotoxins are tightly sorbed to high-affinity clay particles in the gastrointestinal tract, thus decreasing bioavailability, uptake to blood, and potential toxicity. This study investigated the ability of chlorophyll and chlorophyllin-amended montmorillonite clays to decrease the toxicity of ochratoxin A (OTA). The sorption mechanisms of OTA binding to surfaces of sorbents, as well as binding parameters such as capacity, affinity, enthalpy, and free energy, were examined. Chlorophyll-amended organoclay (CMCH) demonstrated the highest binding (72%) and was better than the chlorophyllin-amended hydrophilic clay (59%), possibly due to the hydrophobicity of OTA (LogP 4.7). In silico studies using molecular dynamics simulations showed that CMCH improves OTA binding in comparison to parent clay in line with experiments. Simulations depicted that chlorophyll amendments on clay facilitated OTA molecules binding both directly, through enhancing OTA binding on the clay, or predominantly indirectly, through OTA molecules interacting with bound chlorophyll amendments. Simulations uncovered the key role of calcium ions in OTA binding, particularly in neutral conditions, and demonstrated that CMCH binding to OTA is enhanced under both neutral and acidic conditions. Furthermore, the protection of various sorbents against OTA-induced toxicity was carried out using two living organisms (*Hydra vulgaris* and *Caenorhabditis elegans*) which are susceptible to OTA toxicity. This study showed the significant detoxification of OTA (33% to 100%) by inclusion of sorbents. Organoclay (CMCH) at 0.5% offered complete protection. These findings suggest that the chlorophyll-amended organoclays described in this study could be included in food and feed as OTA binders and as potential filter materials for water and beverages to protect against OTA contaminants during outbreaks and emergencies.

## 1. Introduction

Food contamination has been linked to the onset of many human diseases and poses significant risk to public health in populations across different regions and socioeconomic backgrounds [1] Vulnerable groups include pregnant women, children, elderly populations, and people with weakened immune systems. Environmental pollution, improper storage, and microbial pathogens, among many others, are factors contributing to food contamination. Mycotoxins are naturally occurring low-weight metabolites and biologically active toxins which are produced by various filamentous fungi and play a pivotal role in food contamination globally. The types and levels of mycotoxin are influenced by climatic variations such as temperature, wind, and humidity [2,3]. Their toxic effects include mutagenesis, carcinogenesis, acute or chronic mycotoxicosis, embryotoxicity, immunosuppression, hepatotoxicity, and nephrotoxicity [4].

Ochratoxin A (OTA), a mycotoxin primarily found in dietary products, has been reported as ubiquitous and highly toxic [5,6]. It is produced by *Penicillium* and *Aspergillus* species, principally *P. verrucosum*, *A. niger*, *A. carbonarius,* and *A. ochraceus* [7]. It is predominant in cereals and cereal-based products [8], baby foods [9], dried products [10], beans [11], fruits, wine, and vegetables [12], eggs, pork, fish, poultry, milk and dairy products, herbs, cocoa, coffee, red wine, and tea [13,14]. Exposure to ochratoxin has been associated with many disease conditions and toxicity responses including nephrotoxicity [15], blood–brain barrier damage [16], developmental toxicity and neurotoxicity [17], mutagenicity, and hepatotoxicity [18,19], among others. It is carcinogenic in poultry and rodents [20] and classified as a group 2B carcinogen for humans [21].

Unfortunately, despite these harmful effects elicited by ochratoxin, there is no viable approach for its elimination and detoxification in food and environments where it continues to be abundant, hence the focus of this study. Numerous techniques for ochratoxin detoxification have been developed, but each has drawbacks [22]. For instance, various chemical techniques such as oxidization and alkalization have been suggested, but they may be associated with overt toxicities and potential environmental issues. Physical absorption technology is being suggested as an alternative to these methods because of its low cost, broad application, and recyclability. Natural materials (including bentonite) may be employed as sorbents for the removal of OTA in liquid medium [23]. Furthermore, significant progress has been made in reducing the OTA content of wine and poultry feed through the development of modified bentonite, tri-octahedral bentonite, and similar materials [24,25]. The fact that our organoclay has never been tested for OTA sorption and detoxification highlights the novelty of our work.

One of the most effective approaches to mycotoxin decontamination is the use of viable binders such as activated carbons and edible clays [26]. Clays are incredibly adaptable adsorbent materials that can be used to eliminate pollutants, impurities, and food contaminants [27]. Clays exhibit adsorption mechanisms that enhance attraction and retention of pollutants onto their binding surfaces [28,29,30,31]. Clays occur in a variety of forms, and each of them has special adsorption qualities. Montmorillonite is a type of smectite clay that can have significant swelling qualities and a large surface area. It has been utilized as an adsorbent in many different applications such as the elimination of radioactive substances, organic materials, and heavy metals from soil and water [32]. Calcium and sodium montmorillonite clays have been extensively studied for their binding potency against many chemicals and environmental toxins [28,29,30,31].

Extensive studies from our laboratory have shown that amendment of clays with chlorophyll and other lipophilic compounds improves the binding capacity of clays for lipophilic toxins and enhances their detoxification ability when tested with living organisms. Hence, this study sought to report for the first time the enhanced binding capacity and detoxification ability of modified clays for ochratoxin. Amended organoclays were developed using calcium montmorillonite (CM) and sodium montmorillonite (SM) clay with chlorophyll and chlorophyllin to improve their binding capacity for the lipophilic mycotoxin, OTA. All these materials were generally recognized as safe (GRAS). The study explores the in vitro adsorption endpoints, kinetics, and thermodynamics of surface chemistry interactions and in silico mechanisms of OTA binding to CM and CMCH at both acidic and neutral conditions; simulations investigated the binding properties of both chlorophyll and pheophytin (PHO) molecules. In vivo models were also used to examine the detoxification potency of the amended clays, validate in vitro and in silico results, and establish the proof of concept.

## 2. Results

### 2.1. Physicochemical Properties of the Clays

Table 1 presents the results of the parent clays versus chlorophyll and chlorophyllin-amended clays. Parent sodium montmorillonite (SM) and its derivatives (SMCHin and SMCH) demonstrated a significantly high coefficient of linear expansivity, with SM having a COLE value of 7.0, followed by SMCHin (6.0) and SMCH (1.4). CM-amended sorbents (CMCHin and CMCH) also showed similar COLE values of 1.5 and 1.2, respectively. The pH results revealed that CM- and SM-amended sorbents ranged between neutral and slightly alkaline. All the sorbent materials showed significant bulk density, with parent clays having the highest density of 847.6 kg/m^3^ and 908.4 kg/m^3^ for SM and CM, respectively.

### 2.2. Adsorption Screening

**Figure 1:**
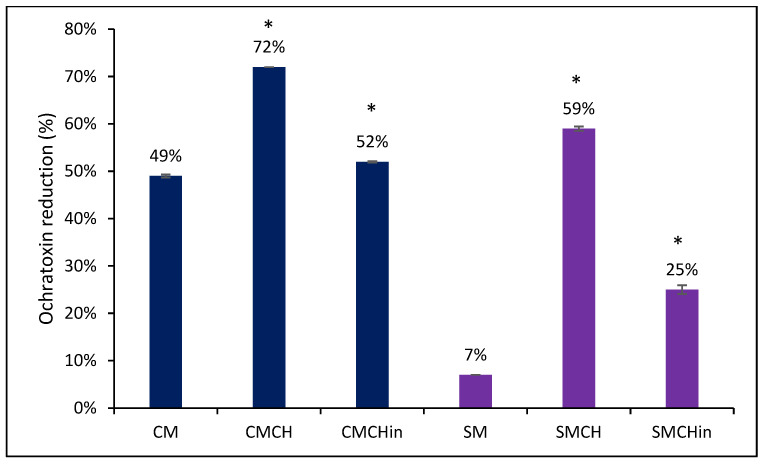
Sorbent screening with reduction percentage of ochratoxin by the sorbents. * *p* < 0.01 when compared to CM or SM. CM: calcium montmorillonite; CMCH: chlorophyll-amended calcium montmorillonite; CMCHin: chlorophyllin-amended calcium montmorillonite; SM: sodium montmorillonite; SMCH: chlorophyll-amended sodium montmorillonite; SMCHin: chlorophyllin-amended sodium montmorillonite.

Figure 1 shows the binding results of OTA onto the surfaces of the amended clays. The parent SM clay slightly sorbed OTA (7%); however, after amendment with chlorophyllin (SMCHin) and chlorophyll (SMCH), the amended clays sorbed OTA by 24.56% and 59.10%, respectively. Furthermore, CM sorbed OTA by 49.56%, but green amendment with chlorophyllin (CMCHin) and chlorophyll (CMCH) significantly enhanced its binding capacity to 52.08% and 71.71%, respectively.

### 2.3. Adsorption Isotherms

**Figure 2:**
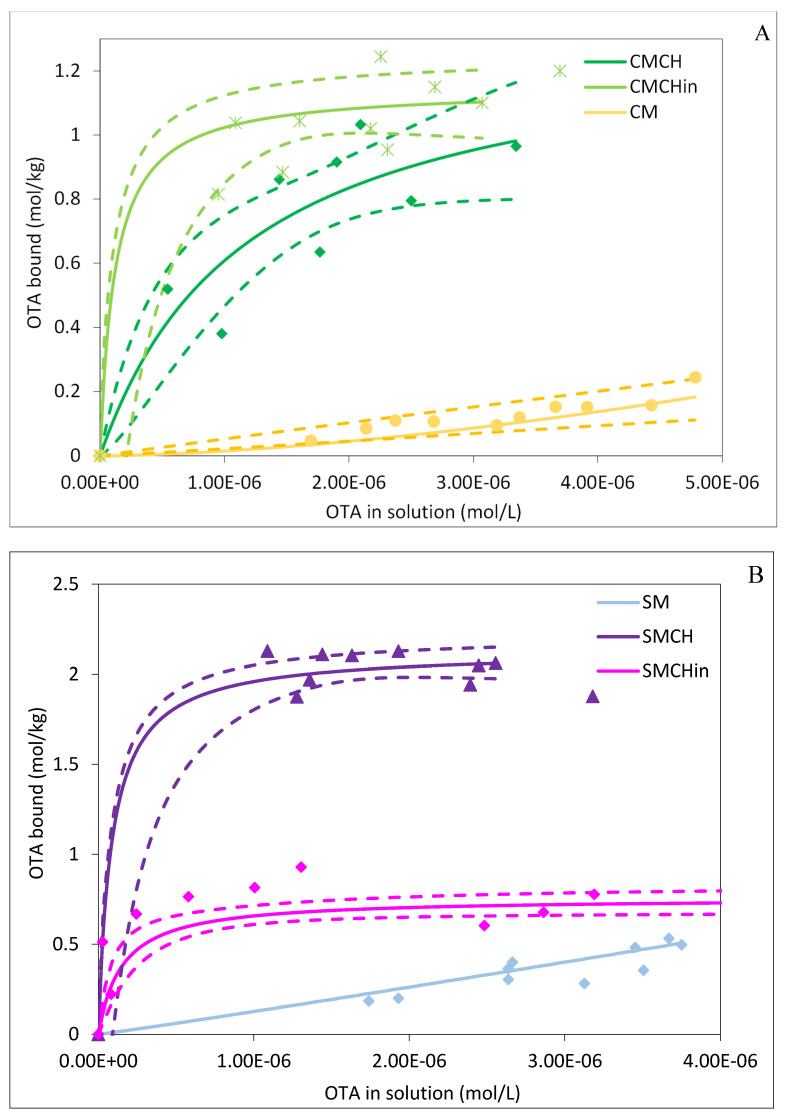
Adsorption isotherms of OTA onto binding surfaces of (**A**) CM-amended clays and (**B**) SM-amended clays at pH 2. CM: calcium montmorillonite; CMCH: chlorophyll-amended calcium montmorillonite; CMCHin: chlorophyllin-amended calcium montmorillonite; SM: sodium montmorillonite; SMCH: chlorophyll-amended sodium montmorillonite; SMCHin: chlorophyllin-amended sodium montmorillonite.

The adsorption isotherms showing the behavior of OTA binding to the surfaces of the clay at pH 2 and 37 °C were plotted in Figure 2A,B. CMCH, CMCHin, SMCH, and SMCHin were plotted using a Langmuir model, and the curved shaped with a plateau indicated that their active sites were homogeneous and saturated. Furthermore, CM and SM were plotted using a Freundlich model and maintained a straight line, suggesting that their active sites were heterogenous and not saturated. All the amended clay sorbents exhibited a significantly high Q_max_ ranging from 0.73 to 2.13, showing sufficient binding sites available for binding. The K_d_ values were at a magnitude of 10^5–7^ as shown in Table 2, which were exceptionally high compared to the literature [33,34] The ∆G of adsorption reactions were negative ranging from −32.98 to −29.74 kJ mol^−1^, indicating that the reactions were thermodynamically favorable and proceeded forward.

### 2.4. Desorption Isotherms

**Figure 3:**
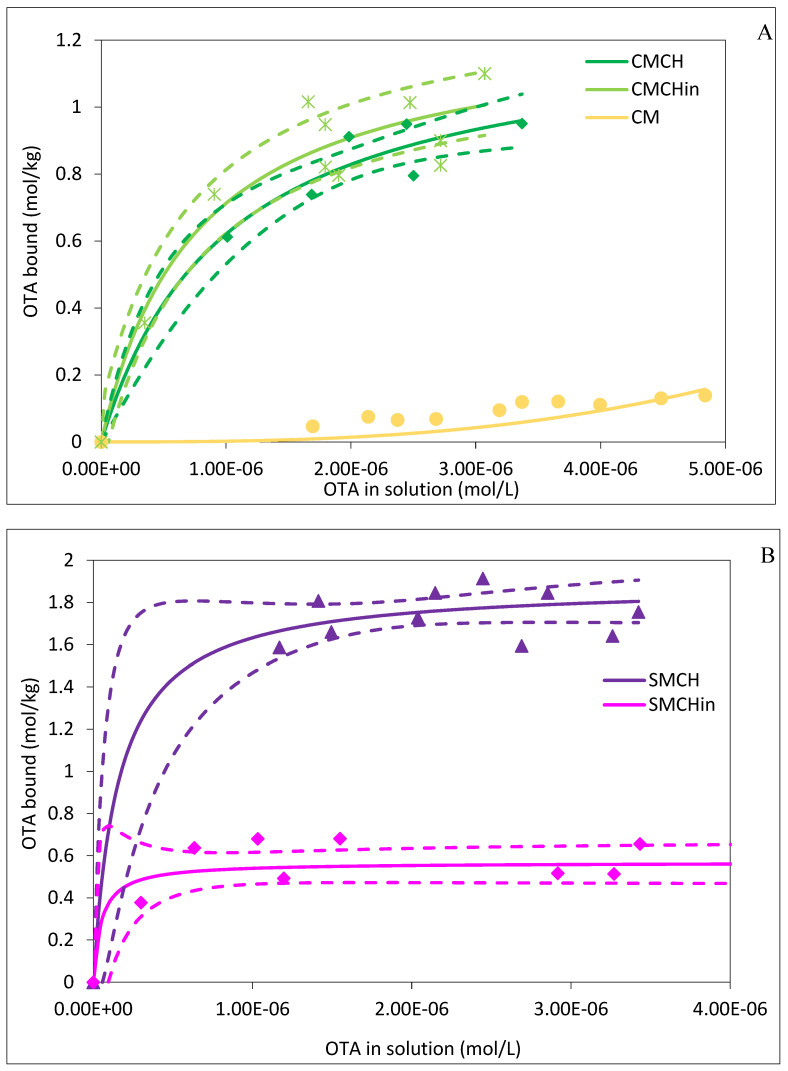
Desorption isotherms of OTA onto binding surfaces of (**A**) CM-amended clays and (**B**) SM-amended clays at pH 6. CM: calcium montmorillonite; CMCH: chlorophyll-amended calcium montmorillonite; CMCHin: chlorophyllin-amended calcium montmorillonite; SM: sodium montmorillonite; SMCH: chlorophyll-amended sodium montmorillonite; SMCHin: chlorophyllin-amended sodium montmorillonite.

The result of the desorption isotherm experiments is shown in Figure 3A,B. A desorption study was carried out to investigate whether the OTA is released from the OTA-bonded sorbent system. This experiment was performed at pH 6 to mimic the pH condition of the human intestine at 37 °C. CMCH, CMCHin, SMCH, and SMCHin demonstrated high Q_max_ and K_d_, indicating a high binding capacity and affinity for OTA (Table 2). The Q_max_ of the desorption reaction of the amended clay sorbents ranged from 0.57 to 1.76 mol kg^−1^, respectively, while their K_d_ ranged in the magnitude of 10^6–7^. The binding percentage after desorption was compared to that of adsorption, which showed a high residual binding of OTA to the sorbent ranging from 73% to 100%. Similarly, the percentage remaining of Q_max_ (∆Q_max_) and K_d_ (∆K_d_) ranged from 16% to 100% (Table 2). However, parent SM showed no Q_max_ and K_d_, suggesting that OTA dissociated completely from the SM complex at pH 6.

### 2.5. Kinetics and Equilibrium of Adsorption

**Figure 4:**
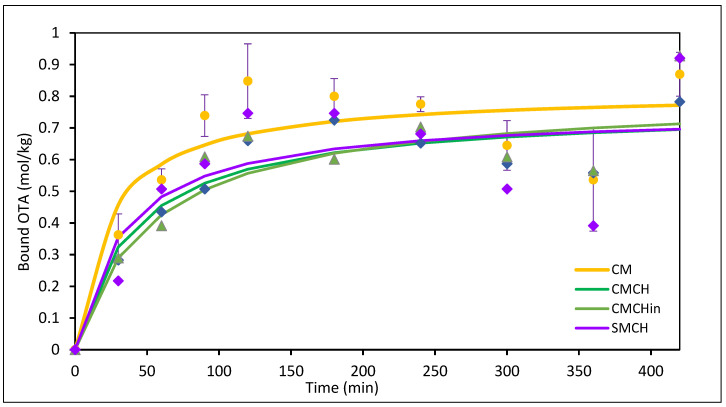
Effect of contact time on the adsorption of OTA. CM: calcium montmorillonite; CMCH: chlorophyll-amended calcium montmorillonite; CMCHin: chlorophyllin-amended calcium montmorillonite; SMCH: chlorophyll-amended sodium montmorillonite.

The kinetics of adsorption of OTA onto the binding surfaces of both the parent and amended clays was elucidated using nonlinear pseudo-first-order, pseudo-second-order, and Elovich models. The pseudo-second-order kinetic model (Figure 4) best characterized the adsorption of OTA onto binding surfaces of the sorbents based on the correlation coefficient value and the consistency between experimental binding capacity (qe, exp) and modeled values (qe, cal). The parameters of the kinetic model are shown in Table 3. CMCHin has the highest binding capacity (qe, exp) value of 0.94, followed by SMCH (0.93) and CM (0.88). SM has a high adsorption rate for the first 2 h of the kinetic study but dissociates completely thereafter, consistent with the Freundlich model.

### 2.6. Thermodynamics of Adsorption

Possible influences of change in temperature and thermodynamic parameters including standard enthalpy change (Δ*H°*) and Gibbs free energy (Δ*G°*) were computed using adsorption isotherms at three different temperatures (4 °C, 18 °C, and 37 °C). The enthalpy (Δ*H°*) of CMCH, CMCHin, SMCH, and SMCHin are −81.80 ± 14.92, −34.56 ± 2.45, −30.15 ± 2.28, and −34.08 ± 11.58 kJ/mol, respectively. It is noteworthy that the amended clays have much improved thermodynamic parameters compared to their parent.

### 2.7. Effect of OTA on Hydra and Detoxification Efficacy of Amended Clays

**Figure 5:**
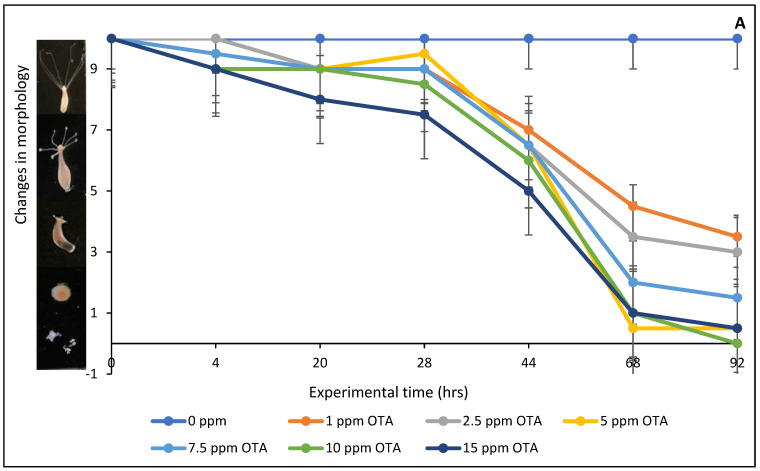

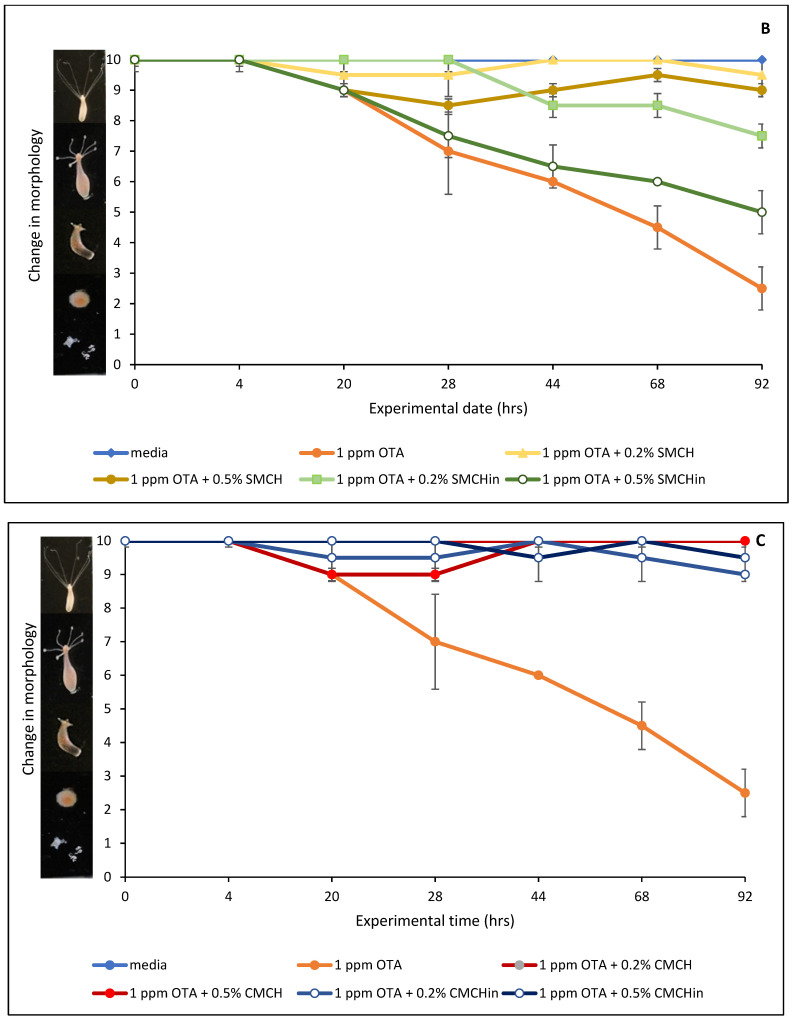
Toxicity effects of OTA exposure to hydra (**A**), protection with SM-derived sorbents (**B**), and CM-derived sorbents (**C**). CM: calcium montmorillonite; CMCH: chlorophyll-amended calcium montmorillonite; CMCHin: chlorophyllin-amended calcium montmorillonite; SM: sodium montmorillonite; SMCH: chlorophyll-amended sodium montmorillonite; SMCHin: chlorophyllin-amended sodium montmorillonite.

The result of in vivo toxicity evaluation of OTA on hydra is presented in Figure 5A. Exposure to OTA within the range of 1 to 15 ppm demonstrated severe toxicity to hydra morphology. Specifically, OTA at various concentrations of 1, 2.5, 5, 7.5, 10, and 15 ppm caused between 65 and 95% damage to the morphology of the hydra. It is noteworthy that the hydra exposed to 10 and 15 ppm of OTA totally disintegrated after 68 h of exposure. Treatment with 0.2% and 0.5% of the montmorillonite-clay-based sorbents significantly protected the hydra colony against OTA toxicity, as shown in Figure 5B,C. Specifically, SM-amended clays (SMCH and SMCHin) at 0.2% and 0.5% inclusion exhibited protection ranging from 33% to 93%, while the CM-amended clays (CMCH and CMCHin) offered protection ranging from 87% to 100%. Remarkably, CMCH at both 0.2% and 0.5% inclusion offered complete (100%) protection.

### 2.8. Effect of OTA on Caenorhabditis Elegans and Detoxification Efficacy of Amended Clays

**Figure 6:**
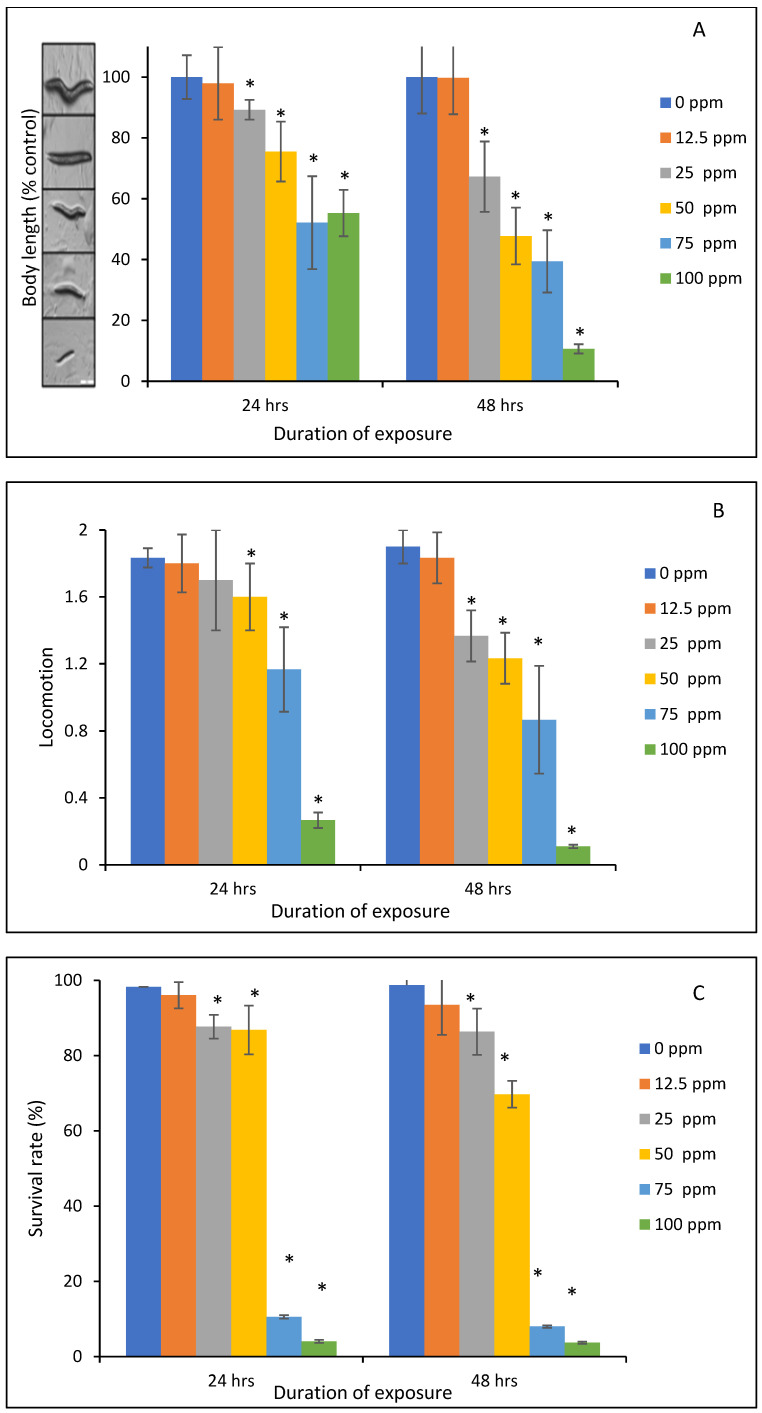
Effect of toxicity of OTA on the body length (**A**), nose touch response (**B**), and survival rate of *Caenorhabditis elegans* after 24 h and 48 h of exposure (**C**). Data represent the average value from triplicate analysis  ±  the standard deviation. * indicates a significant difference (*p*  ≤  0.05) compared to the vehicle control group.

**Figure 7:**
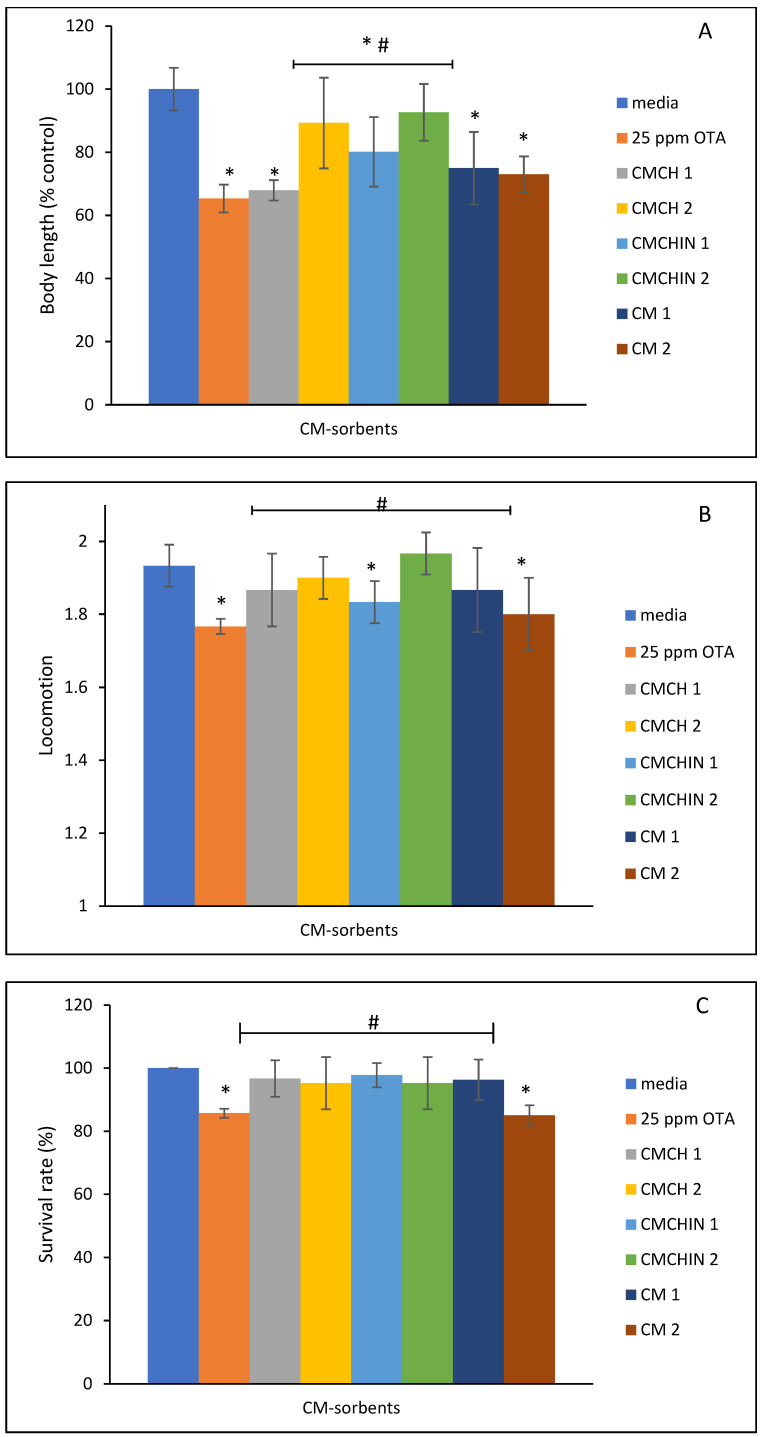

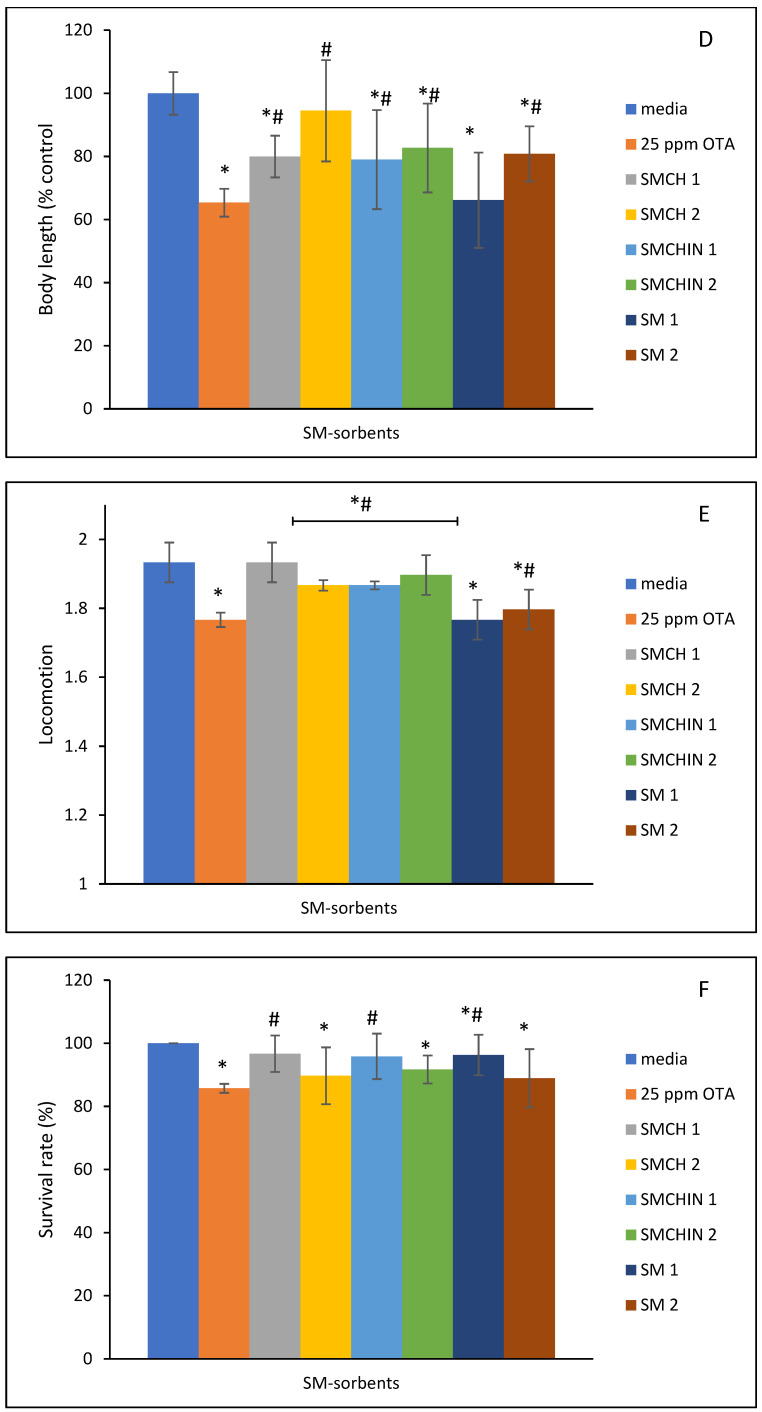
Protective effect of parent clays and amended clays against OTA toxicity on the body length (**A**,**D**), nose touch response (**B**,**E**), and survival rate of *Caenorhabditis elegans* (**C**,**F**). Data represent the average value from triplicate analysis  ±  the standard deviation. * indicates a significant difference (*p*  ≤  0.05) compared to the vehicle control group. # indicates a significant difference (*p*  ≤  0.05) compared to the OTA-alone group. 1: 0.2% and 2: 0.5% clay inclusions; CM: calcium montmorillonite; CMCH: chlorophyll-amended calcium montmorillonite; CMCHin: chlorophyllin-amended calcium montmorillonite; SM: sodium montmorillonite; SMCH: chlorophyll-amended sodium montmorillonite; SMCHin: chlorophyllin-amended sodium montmorillonite.

Figure 6 and Figure 7 reveal the results of the toxic effects of OTA on *Caenorhabditis elegans* and protection by the sorbents. As shown in Figure 6A, both 24 and 48 h exposure to OTA from 12.5 to 100 ppm affected the body length of the worms. The relative body length of nematodes in all concentrations tested in this study was decreased compared to the medium control group, which was adjusted to 100% (Figure 6A). Interestingly, 25 ppm of OTA decreased body length to 89% and 67% of the control at 24 h and 48 h exposure, respectively, indicating marked growth inhibition. Similarly, the movement ability of the worms was significantly retarded following exposure to varying concentrations of OTA (Figure 6B). Furthermore, the survival rate was affected in a concentration-dependent pattern (Figure 6C). Particularly, the lethality of worms at levels below 12.5 ppm was insignificant, whereas the lethality rate increased as OTA exposure level increased. At 25 ppm, OTA significantly decreased the survival rate of nematodes to 87% (*p*  <  0.05). At 75 and 100 ppm, there is a complete mortality of the worms both at 24 and 48 h of exposure. Due to these observations, 25 ppm of OTA was used in the sorbent treatment study to validate sorbent efficacy.

Treatment with 0.2% and 0.5% of parent clays and green sorbents for 24 and 48 h protected worms from the toxicity of 25 ppm OTA with a marked increase in body length (Figure 7A, *p*  <  0.05), enhanced the response of the worms to nose touch (Figure 7B, *p*  <  0.05), and increased survival rates (Figure 7C, *p*  <  0.05). The protection offered by the organoclay is higher than the parent.

### 2.9. Molecular Dynamics (MD) Simulations of OTA onto the Binding Surfaces of the Green-Amended Clays

**Figure 8:**
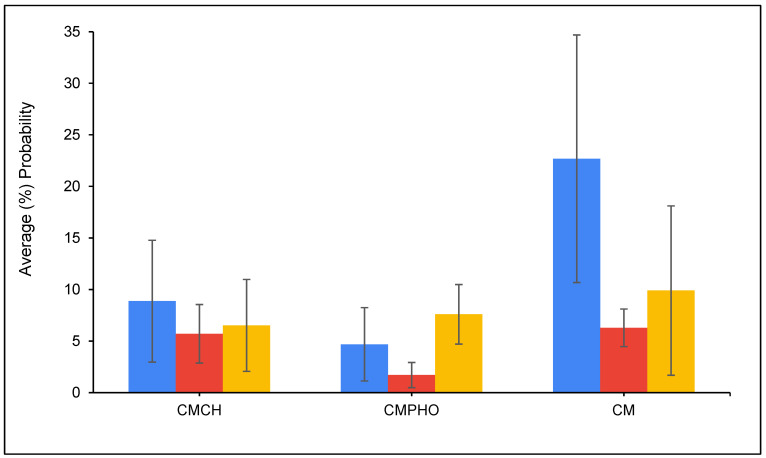
Average (%) probability of interaction between OTA molecules in the presence of CMCH, CMPHO, and CM. Blue corresponds to acidic conditions; red and yellow correspond to neutral conditions, simulating monoanionic and dianionic OTA, respectively. Average values are calculated from triplicate runs. Error bars denote standard deviation values calculated from the triplicate runs.

**Figure 9:**
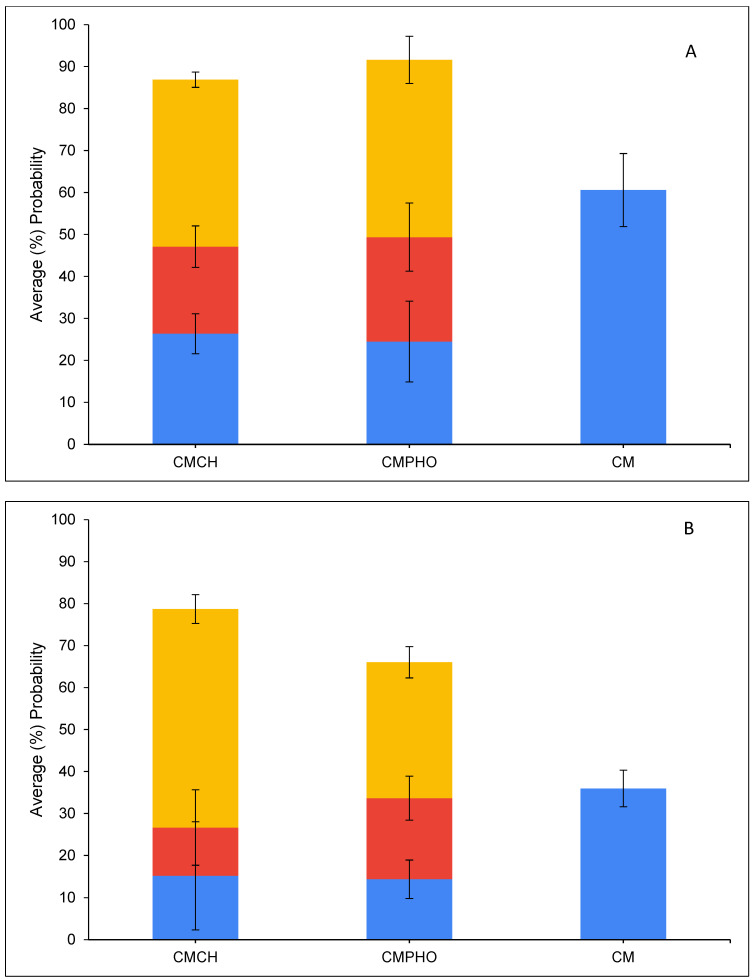

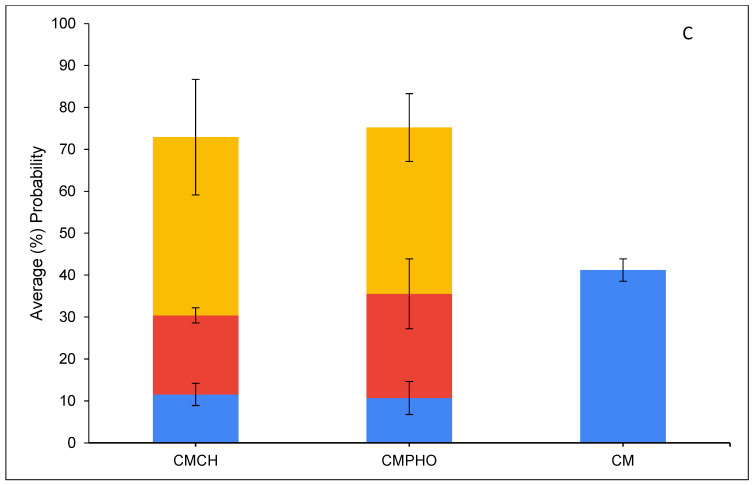
Average (%) probability of OTA molecules interacting with CMCH, CMPHO, and CM in acidic condition (**A**) and neutral condition (**B**,**C**), simulating monoanionic and dianionic OTA, respectively. Direct, direct-assisted, and indirect-assisted interactions are shown in blue, red, and yellow, respectively. Average values are calculated from triplicate runs. Error bars denote standard deviation values calculated from the triplicate runs.

**Figure 10:**
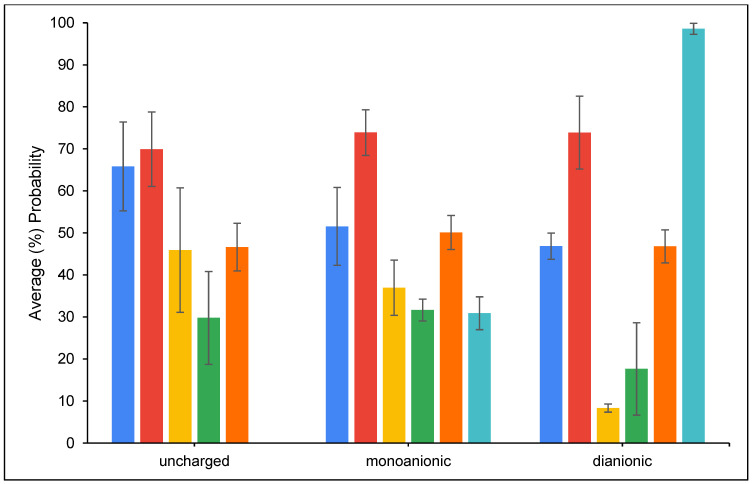
Average (%) probability of CM participating in interactions with different OTA groups (defined in Figure S2). Group 1 corresponds to blue, group 2 corresponds to red, group 3 corresponds to yellow, group 4 corresponds to green, and group 5 corresponds to orange. Additionally, the average (%) probability of CM-bound OTA molecules interacting with calcium is shown in cyan. The results were normalized, i.e., they were calculated given an interaction between OTA and CM. Values correspond to parent (CM) and amended clays (CMCH and CMPHO) in acidic conditions (left), as well as neutral conditions (middle) and (right), of simulations including monoanionic and dianionic OTA, respectively. Average values are calculated from triplicate runs. Error bars denote standard deviation values calculated from the triplicate runs.

Figure 8 presents the average percentage probability of an OTA molecule interacting with another OTA molecule. The results showed that OTA molecules have a higher tendency to interact with each other in the parent clay (CM) simulations compared to chlorophyll- or pheophytin-amended clay (CMCH and CMPHO, respectively) simulations. Overall, OTA molecules have a relatively high tendency to (directly) bind to CM, which ranges from ~35–40% for OTA binding in monoanionic and dianionic states in complex with CM at neutral conditions to ~60% for uncharged OTA in complex with CM at acidic conditions, as shown in Figure 9 (CM). Figure 10 presents the normalized average percentage probability of interactions formed by different OTA groups either in complex with CM and/or calcium. The direct binding of uncharged OTA to CM at both acidic and neutral conditions can be predominantly attributed to contacts formed by non-polar (i.e., aromatic and hydrophobic) groups (as defined in Figure S2) with CM (Figure 10). Interactions between OTA hydrophilic groups 3 and 4 with CM are reduced for the monoanionic state and even more for the dianionic state (Figure 10), while there is an increase in interactions with calcium ions, a small portion of which are also associated with calcium-mediated interactions with CM (Figure 10). This, based on additional visual inspection, can be attributed to the higher tendency of carboxyl groups (COO^−^/COOH) to be solvent-exposed and also interact with calcium ions, which may be either unbound or bound to the clay, especially when OTA is in its dianionic state (Figure S5 and Figure 10). In the cases where calcium ions are bound to the clay, calcium ions mediate interactions between CM and the hydrophilic groups.

**Figure 11:**
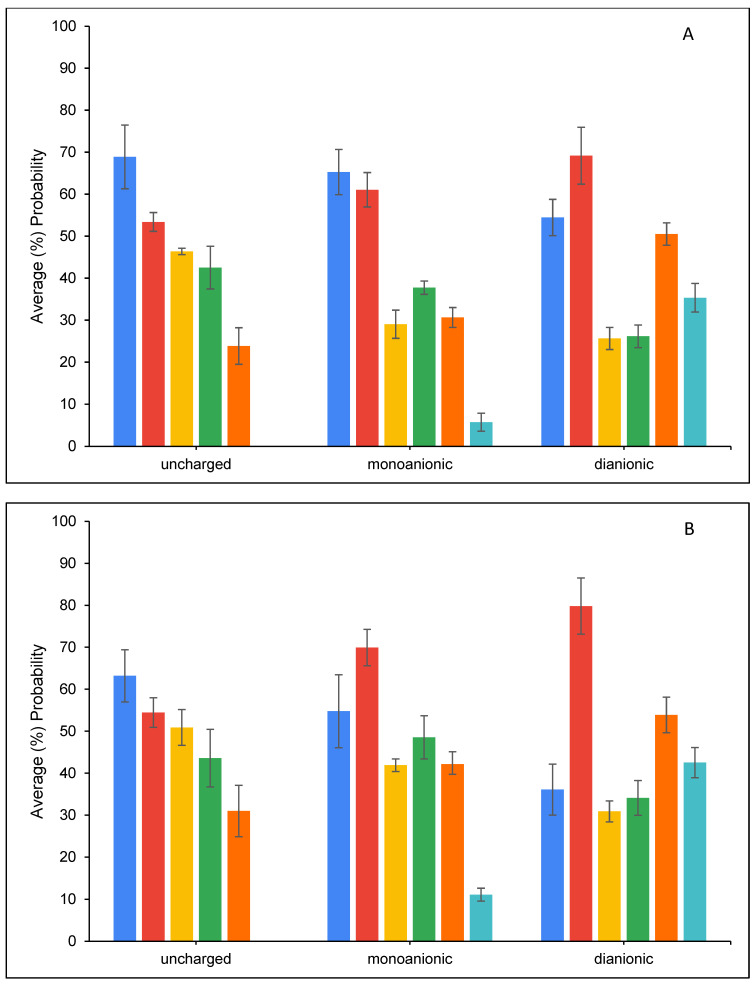
Average (%) probability of CMCH (**A**) and CMPHO (**B**), respectively, participating in interactions with different OTA groups (defined in Figure S2). Group 1 corresponds to blue, group 2 corresponds to red, group 3 corresponds to yellow, group 4 corresponds to green, and group 5 corresponds to orange. Additionally, the average (%) probability of CMCH-bound (**A**) or CMPHO-bound (**B**) OTA molecules interacting with calcium is shown in cyan. The results were normalized, i.e., they were calculated given an interaction between OTA and CMCH and CMPHO. Values correspond to systems in acidic conditions (left), as well as neutral conditions (middle) and (right), of simulations including monoanionic and dianionic OTA, respectively. Average values are calculated from triplicate runs. Error bars denote standard deviation values calculated from the triplicate runs. All values shown above are normalized over the total number of interactions per system.

The presence of amendments in either CMCH or CMPHO enhances binding of OTA, both through indirect and direct assisted interactions (Figure 9), which is accompanied by a slight reduction in probability of OTA molecules interacting with each other as well as directly binding to the clay without any interaction with chlorophyll or pheophytin (Figure 8). The degree of enhancement seems to be overall similar across different cases, considering neutral versus acidic conditions (Figure 9). The slightly overall higher average percentage probability of OTA at both CMCH and CMPHO at acidic conditions in comparison to neutral conditions can be primarily attributed to the enhanced direct binding of the uncharged OTA to the acidic clay (Figure 9). There are not any particular differences between CMCH and CMPHO overall, except for the fact that the CMCH enhancement is of a higher degree than CMPHO enhancement for monoanionic OTA under neutral conditions; nevertheless, this is somehow counterbalanced by the slightly higher degree of enhancement in CMPHO compared to CMCH in the cases of uncharged and dianionic OTA (Figure 9). According to visual inspection of the simulations, there is no clear tendency for particular unique modes of interaction between the OTA and chlorophyll or pheophytin, but rather, there is a tendency for the non-polar groups of uncharged, monoanionic, and dianionic OTA to interact especially with the aliphatic tail but also with non-polar groups of the head of chlorophyll or pheophytin. To further examine which groups of OTA interact with chlorophyll and pheophytin, we calculated the normalized average percentage probability of interactions formed by different OTA groups (as defined in Figure S2) in complex with amending molecules, chlorophyll or pheophytin, in the CMCH and CMPHO, respectively, as well as calcium ions (Figure 11). Interactions between OTA hydrophilic groups 3 and 4 with amended chlorophyll or pheophytin molecules are reduced for the monoanionic state and even more for the dianionic state (Figure 11), while there is an increase in interactions with calcium ions (Figure 11). The aforementioned analysis in combination with visual inspection of the simulations provided additional mechanistic understanding of OTA binding to CMCH and CMPHO. As mentioned above for CM, in CMCH and CMPHO, analogously here, this can be attributed to the higher tendency of carboxyl groups (COO^−^/COOH) to be solvent-exposed and also interact with calcium ions, which may be either unbound or bound to the clay, especially when OTA is in its dianionic state. Additionally, hydrophobic group 2 and chlorine (group 5), which are on the opposite site of OTA (COO^−^/COOH), have higher tendencies to interact with non-polar moieties of chlorophyll- or pheophytin-amended molecules. This occurs more in monoanionic and especially dianionic states in neutral conditions, compared to the uncharged state in acidic conditions. Group 1 has an overall tendency to interact with the non-polar moieties of chlorophyll or pheophytin through aromatic–hydrophobic interactions. Interestingly, interactions between aromatic group 1 and chlorophyll or pheophytin tend to decrease in neutral conditions, especially when OTA is in a dianionic state, which can be somehow attributed to its proximity to carboxyl groups coordinating with calcium ions; this occasionally leads to the formation of cation–π interactions between the aromatic group and calcium ions according to visual inspection.

**Figure 12:**
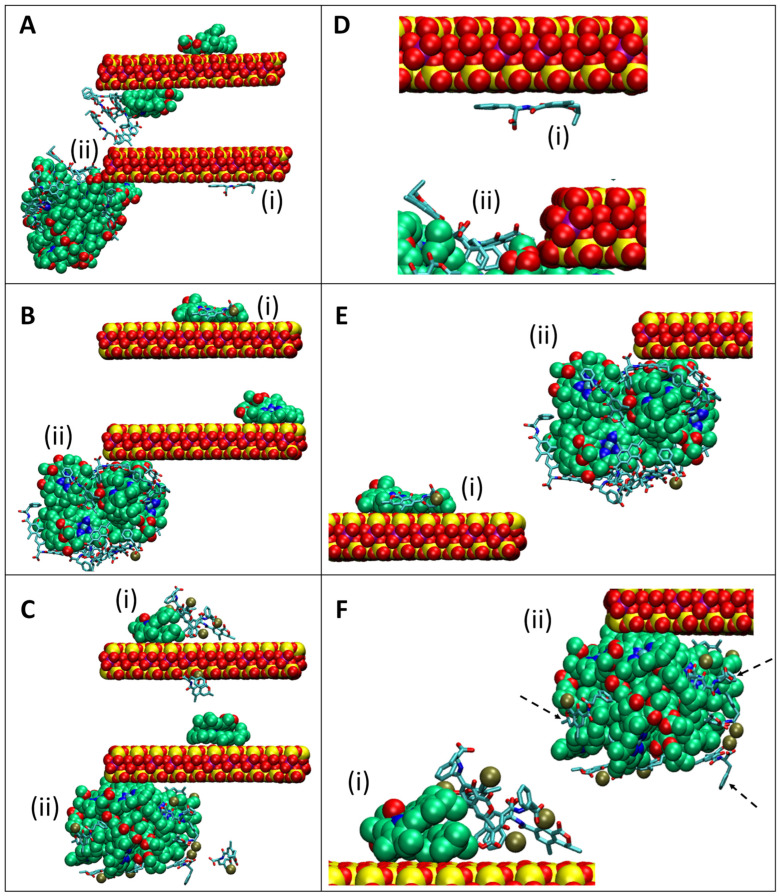
Simulation snapshots of CMCH in complex with OTA in (**A**) acidic conditions, as well as (**B**,**C**) neutral conditions, investigating OTA in monoanionic and dianionic states, respectively. Panels (**D**–**F**) show zoomed-in representation of particular interactions, marked as (i) and (ii), that occur within panels (**A**–**C**), respectively. CM, chlorophyll, and calcium are shown in vdW representation, while OTA is shown in licorice representation. Atoms are colored by atom type, except for carbon atoms of chlorophyll in green and calcium in tan. Calcium ions that are at a greater distance than 3.5 Å from all OTA molecules were omitted. Hydrogen atoms are also omitted for clarity.

Figure 12 presents molecular graphics images of the last (100 ns) simulation snapshots for selected simulation runs of OTA interacting with CMCH in acidic conditions (Figure 12A), monoanionic OTA interacting with CMCH in neutral conditions (Figure 12B), and dianionic OTA interacting with CMCH in neutral conditions (Figure 12C), respectively. Figure 12D–F show zoomed-in representations of Figure 12A–C, respectively, emphasizing particular modes of interaction. Figure 12A shows a chlorophyll-bound molecule within the interlayer contributing to the binding of direct-assisted OTA on the top layer and an indirect-assisted OTA as well as an additional direct OTA binding to the bottom layer; notably, the three aforementioned OTA molecules form a cluster which synergistically contributes to their binding. Additionally, Figure 12A shows an individually bound OTA molecule, which according to its zoomed-in representation in Figure 12D(i), directly binds lying flat on the surface, and thus, all chemical groups (as defined in Figure S2) interact with CM; yet, it is important to note that the carboxyl group is pointing away from the clay exposed to the solvent. This comprises a representative example of uncharged OTA directly binding under acidic conditions. Also, Figure 12A shows a cluster of chlorophyll-bound molecules which contribute to a combination of direct- and indirect-assisted interactions of OTA molecules. Particularly, Figure 12D(ii) shows a zoomed-in representation of two OTA molecules in a direct-assisted interaction with a cluster of chlorophyll-bound molecules at which the non-polar groups of OTA are in contact with the hydrophobic groups of chlorophyll, while the polar groups of OTA are exposed to the solvent. Figure 12E(i,ii) show zoomed-in representations of Figure 12B, comprising two examples of OTA (monoanionic state) interacting with CMCH in neutral conditions. Figure 12E(i) shows an example of an OTA molecule participating in a direct-assisted interaction in which the OTA molecule interacts with clay through its chlorine atom. In this case, OTA and chlorophyll interact both through their polar and non-polar moieties, while a calcium ion mediates an interaction between the clay and OTA’s carboxyl group. Figure 12E(ii) presents an example of a chlorophyll-bound cluster attracting OTA molecules, such that their hydrophobic and aromatic groups are predominantly oriented towards the interior, forming non-polar contacts with a cluster of chlorophyll-bound molecules, while their hydrophilic groups are pointing primarily toward the exterior. Additionally, two negatively charged carboxyl groups of a pair of two neighboring OTA molecules interact with a calcium ion. Figure 12C presents an example of a chlorophyll molecule and a cluster of chlorophyll-bound molecules at the exterior of the top and bottom layers, respectively, contributing to a combination of direct- and indirect-assisted interactions with OTA molecules which individually coordinate with calcium ions; zoomed-in representations are presented in Figure 12F(i,ii). Figure 12F(i) shows four OTA molecules (from a cluster of five OTA molecules) participating in indirect-assisted (top molecule), direct-assisted (middle-front and middle-back molecules), or direct interactions (right molecule). The interactions predominantly involved OTA and chlorophyll non-polar groups. Each of the four OTA molecules is coordinated with a calcium ion attracted to its negatively charged hydrophilic group, and one calcium ion in particular mediated interactions of the middle front OTA molecule with the clay. In Figure 12F(ii), the top-right, middle-left, and bottom-right OTA molecules (shown via arrows) interact with chlorophyll via their hydrophobic moieties and the chlorine atom, while their hydrophilic groups are facing toward the solvent and are coordinated with a calcium ion; the main difference between the three lies in the fact that in the bottom right case, the aromatic ring is more exposed to the solvent in comparison to the two other cases, in which it is buried.

## 3. Discussion

Some environmental toxins and food contaminants have been shown to exhibit preferential binding to clays [35]. In this study, the binding capacity of parent clays (CM and SM) was improved through the addition of chlorophyll and chlorophyllin to their interlayer. This resulted in a notable increase in the hydrophobicity of the clay surfaces with the chlorophyll-amended clay.

The preliminary screening of the binding ability of the clays demonstrated that all the amended clays exhibited a significantly higher binding percentage (ochratoxin reduction) than their respective parent clays. This finding potentiates green clay strategies as viable applications to enhance the binding capacity of parent clays. Factors such as pH, structural morphology of the clay, solubility, speciation, and charge of pollutants are crucial to adsorption investigations. As shown in the result of this study, the pH_pzc_ of all the amended clays was greater than 2, indicating that the clays’ surfaces tend to be positively charged at pH 2 due to the protonation of the hydroxyl groups, thus facilitating electrostatic interactions [22].

Understanding the relationship between OTA and the various surfaces of the adsorbents requires the use of adsorption isotherms. Adsorption process efficiencies, capacities, and equilibrium conditions can all be ascertained with the aid of these isotherms. They can improve the theoretical understanding of adsorption, which allows for improved modeling and predicting capacities [36]. Adsorption isotherms were used in this investigation as shown in the results. The correlation coefficient (r^2^) indicated that the Langmuir adsorption isotherm was the best-fitting model for all the amended clays (CMCH, CMCHin, SMCH, and SMCHin). This implies that OTA molecules form a single layer on the adsorbent surfaces through a monolayer adsorption mechanism with no further contact between OTA molecules during the adsorption process [37]. On the other hand, the parent clays (CM and SM) fit the Freundlich isotherm, which presumes multilayer adsorption. According to Mukherjee et al. [38], the 1/n values may be used as a predictor of the viability and favorability of adsorption, providing information on the adsorption intensity and surface heterogeneity. Chernomorova et al. [39] documented that 1/n indicates the types of isotherms: irreversible (1/n = 0), favorable (0 < 1/n < 1), and unfavorable (1/n > 1). The 1/n values of CM and SM in this investigation were found to be between 0 and 1, indicating a successful adsorption process.

Thermodynamic studies were carried out to investigate the effect of changes in temperature on the adsorption of OTA on the various sorbents. Usually, temperature influences adsorption processes by altering the molecular energy and adsorption capability. Temperature can change the thermodynamic equilibrium of the adsorption reactions and has an impact on the desorption process as well [40]. Three temperatures (4 °C, 18 °C, and 37 °C) were examined in this investigation, and an obvious trend of temperature-reliant changes was noted. The adsorption systems exhibited the maximum adsorption capacities at 4 °C, suggesting that this temperature enhances OTA adsorption onto the surfaces of the sorbents; this indicated that larger adsorption capabilities were attained at lower temperatures. But as the temperature increased to 18 °C and 37 °C, there was a decline in the adsorption capacity. Temperature could promote adsorption processes by enhancing the kinetic energy of adsorbate molecules and causing more regular and vigorous collisions between the adsorbent and the adsorbate. As a result, the adsorbent surface is reached by the adsorbate molecules more effectively and at a faster pace due to enhanced molecular diffusion. Temperature can also increase the solubility of adsorbates and reduce the interactions between adsorbates, thus resulting in larger concentrations of the absorbate at the adsorbent surface [41]. Nevertheless, the precise influence of temperature on adsorption may change based on the nature of the adsorbent–adsorbate system.

One factor that influences adsorption performance is the contact time of the interaction between the molecule of adsorbate (OTA) and adsorbents [42]. As demonstrated in the results, the elimination efficacy of OTA rose with time until equilibrium was reached. Within the first 30 min, OTA was adsorbed quickly; however, as the adsorbents’ active sites were saturated, the adsorption process became steady. In the first 120 min, the adsorption rate for OTA was likewise quick. Following the equilibrium time, the rate of adsorption stabilized. Two events could happen at equilibrium: (i) the reaction process may remain at equilibrium, and (ii) due to the poor interaction between the adsorbent and adsorbate, desorption may occur as a result of further agitation. The huge surface area that was available for adsorption was the cause of the high rate of adsorption at the initial stage of the reaction. As the active sites were occupied and the remaining empty sites could not be occupied due to the repulsive interaction between the solute molecules, the process rate became constant over time [43].

The *Hydra vulgaris* experiment is a well-known in vivo model and thus is used to validate in vitro studies and establish the safety and effectiveness of sorbent products [29]. The result of this current study demonstrated that exposure to OTA in hydra colonies at varying concentrations resulted in different levels of toxicity, including death of the organisms at higher concentrations. Previous studies have reported OTA oxidative-stress-mediated toxicities in hydra [44]. Nevertheless, the inclusion of the amended clays offers significant protection, with CMCH offering total protection against the toxicity of OTA up to 92 h of exposure. OTA must remain bound to the sorbents in the gastrointestinal system for a sufficient time in order to be eliminated from the body. This physiological process normally takes up to 24 to 48 h after ingestion [45]. Studies have also shown that peristalsis and gastrointestinal transit durations may vary between people and sexes [45]. OTA absorption through the lumen can be prevented by the sorbent-based reduction in the bioavailability during physiological digestion in humans and animals, according to an assessment during this crucial period in hydra [46,47]. It validated that toxicity can be prevented for a longer time. Thus, this study suggested that these amended clays could be viable enterosorbents for the detoxification of OTA.

The toxicity testing procedures for *Caenorhabditis elegans* are well established, and the model is a dependable toxicological tool that can detect a broad variety of pollutants at quantities that are relevant to the environment [48,49]. In line with the literature, our initial *C. elegans* investigations using montmorillonites and other comparable smectite clays have shown that the nematodes were not negatively affected by the addition of these clay materials at 0.2 and 0.5% [50,51]. The rate of survival and locomotion of *C. elegans* were negatively impacted when they were exposed to different OTA concentrations, indicating that OTA inhibited the growth of *C. elegans*. The results of this investigation are consistent with those of a prior study, which has shown that long-term exposure to mycotoxin lowers *C. elegans* lifespan and frequency of movement, along with altering ageing markers and inducing ROS levels [52]. The degree of improved survival rate and body length resulting from the protection provided by the sorbents was similar. Significantly, both the 24 and 48 h treatment with sorbent inclusions produced this notable protection. Previous dosimetry and time course investigations suggest that a longer treatment period and a higher incorporation of sorbents may lead to a higher protection rate [51,53].

Within the simulations, OTA molecules have a higher tendency to interact with each other in the CM (control) simulations compared to chlorophyll- or pheophytin-amended clay (CMCH and CMPHO, respectively) simulations. According to visual inspection, uncharged OTA was found to aggregate more compared to monoanionic and dianionic OTA molecules in the CM (control) simulations, with dianionic OTA being more prone to interact with each other and, as a result, more prone to aggregate than monoanionic OTA (Figure S5). The clustering of OTA is predominantly driven by non-polar (i.e., aromatic and hydrophobic) interactions and hydrogen bonding to a significantly lesser extent; additionally, the slightly higher probability of dianionic OTA molecules to interact with each other (Figure 8) could possibly be attributed to the capacity of each single OTA molecule to form a “neutral” entity in coordination with calcium (Figure S5). Notably, the higher percentage of direct binding for the dianionic compared to the monoanionic state in the CM (control) simulations could be attributed to the fact that dianionic OTA can be neutralized via coordination with a single calcium ion, in contrast to monoanionic OTA, in which a pair of two molecules is necessary. Overall, it is important to highlight that calcium ions, which are part of the system of the montmorillonite clay, contribute to the mechanisms of OTA binding to CM, CMCH, and CMPHO, particularly in neutral conditions. Calcium ions primarily contribute to interactions with OTA carboxyl groups, facilitating stabilization of non-polar interactions between OTA and amended chlorophyll or pheophytin. Additionally, calcium ions can in some cases mediate interactions between clay and OTA.

## 4. Conclusions

Overall, this study demonstrated that the amendment of parent clays (CM and SM) with chlorophyll and chlorophyllin improved the binding capacity for OTA at physiological pHs (pH 2 and 6) for the gastrointestinal system. This study also established that the binding between OTA and amended clays was tight and reliable for more than 48 h, suggesting that the amended clays could serve as viable enterosorbents to facilitate the elimination and detoxification of OTA in humans and animals. In silico simulations supported the in vitro findings and demonstrated that amendment of CM with chlorophyll enhanced binding of OTA through both indirect- and direct-assisted interactions, in both acidic and neutral conditions. Furthermore, simulations demonstrated that OTA interactions with chlorophyll involve predominantly the non-polar groups of the two molecules, while additionally elucidating the key role of calcium ions. Montmorillonite clays and chlorophyll have been reported to be safe for human consumption when included in the diet. Importantly, these green clays can serve to reduce OTA exposure in animals and humans from contaminated food and water. Clinical trials are warranted to validate the efficacy and safety of these novel clays.

## 5. Materials and Methods

### 5.1. Chemicals and Reagents

Ochratoxin A (OTA) was purchased from Sigma Aldrich (Saint Louis, MO, USA). Chlorophyllin and sulfuric acid (H_2_SO_4_, 95–98%) were purchased from Aldrich Chemical Co. (Milwaukee, WI, USA). Chlorophyll was purchased from Santa Cruz Biotechnology (Dallas, TX, USA). High-pressure liquid chromatography (HPLC)-grade acetonitrile, and pH buffers (4.0, 7.0, and 10.0) were purchased from VWR (Atlanta, GA, USA). Ultrapure deionized water (18.2 MX) was generated in the lab using an Elga™ automated filtration system (Woodridge, IL, USA) and was used in all experiments. Sodium montmorillonite (SM) was obtained from the Source Clay Minerals Repository at the University of Missouri–Columbia with cation exchange capacity equal to 75 cmol kg^−1^. Calcium montmorillonite (CM) was obtained from Engelhard Corp. (Cleveland, OH, USA) with cation exchange capacity equal to 97 cmol kg^−1^, an external surface area of approximately 70 m^2^ g^−1^, and an average total surface area as high as 850 m^2^ g^−1^ [54]. The generic formula representing both clays is (Na,Ca)_0.3_(Al,Mg)_2_Si_4_O_10_(OH)_2_·nH_2_O. Samples contain some calcite, sanidine, quartz, and mica as impurities, as well as mesopores of approximately 5 nm in diameter [55].

### 5.2. Synthesis and Amendment of Multicomponent Clays

CM and SM clays were amended with water-insoluble chlorophyll to produce organoclays, CMCH and SMCH, respectively, and with water-soluble chlorophyllin to produce hydrophilic CMCHin and SMCHin, respectively, at 150% cation exchange capacity. Briefly, CM or SM at 5% *w*/*w* in pH 4.2 water were stirred vigorously with chlorophyll or chlorophyllin for 24 h to facilitate oversaturation of exchangeable sites with chlorophyll or chlorophyllin. The suspensions were then centrifuged at 3000× *g* for 20 min and thereafter washed with distilled water. The washing process was repeated 3 times, and all samples were dried in a desiccator before being pulverized and passed through a 150 µm sieve. The characterization of these clays including particle size, zeta potential, expansibility in water, Fourier-transform infrared spectroscopy (FTIR, IRPrestige-21, Shimadzu, Japan), X-ray powder diffraction (XRD, Bruker D8 Endeavor, Germany), scanning electron microscopy (SEM, JSM-7500F, JEOL, Peabody, MA, USA), lipophilicity, and light sensitivity, demonstrating the stability of chlorophylls in clay interlayers that have been previously reported from our laboratory [29,56]. Physicochemical properties such as pH, pHpzc, bulk density, coefficient of expansibility in water, moisture content, and hydrophobicity were carried out as previously described.

### 5.3. Screening of Clays

OTA stock solution was prepared by dissolving its pure powder in acetonitrile at 1250 ppm (µg/mL) concentration. In order to validate the OTA detection method, a standard solution was prepared in deionized water at concentrations ranging from 0.01 ppm (µg/mL) to 10 ppm. Standard curves with linearity (r^2^ > 0.98) were constructed using the equation Y = 0.157x + 0.5615. A glass syringe was used to measure the appropriate quantity of the OTA stock solution to make a 1 ppm OTA solution in pH 2 water. The ideal sorbent-to-OTA ratio needed to achieve saturation in isotherm plots served as the basis for selecting this concentration. Each sorbent was introduced at 4 mg/mL into 1.5 mL OTA solution and mixed at 1000 rpm and 37 °C for 2 h to determine the sorbent binding capacity. Thereafter, the mixture was centrifuged at 3000× *g* for 5 min. The quantity of OTA present in the supernatants was determined using UV/Vis spectrophotometry at 212.2 nm.

### 5.4. Sorption Isotherms

The adsorption capacity of sorbents and the effective sorbents dose were determined for 1 ppm OTA. Each sorbent was introduced at 4 mg/mL into a gradient of 1.5 mL OTA solutions ranging from 5% to 100% without adjusting the pH of the solution. Control solutions contained 1.5 mL of blank water (pH 2), OTA, and each sorbent in water, respectively. To simulate the ingestion in the human stomach, these solutions were shaken at pH 2 at 1000 rpm and 37 °C for 2 h. OTA was separated via centrifugation at 3000× *g* for 5 min and determined using UV/Vis spectrophotometry at 212.2 nm. After this, a desorption analysis was carried out. Briefly, each test tube was washed with pH 6 water and spun at 3000 rpm for 5 min to remove unbound OTA. The tubes were made up of 1.5 mL of pH 6 water and shaken at 1000 rpm and 37 °C for 48 h to replicate the mixing condition of the human intestine. Control solutions contained 1.5 mL of blank water (pH 6), OTA, and each sorbent in water, respectively.

In the thermodynamic analyses, to determine the enthalpy and Gibbs free energy, the mixtures containing different sorbent/OTA combinations and controls were shaken at 1000 rpm at three different temperatures: 4 °C, 18 °C, and 37 °C. Following this, OTA was extracted from the sorbent/OTA mixtures using centrifugation for 5 min at 3000× *g*. The OTA concentration in the supernatants was measured at 212.2 nm using UV/Vis spectrophotometry.

### 5.5. Adsorption Kinetics

In this investigation, adsorption kinetic models were used to assess the capability of various sorbents to bind OTA over an exposure period of 420 min. Briefly, each sorbent at 4 mg/mL was mixed separately with 1 ppm OTA in pH 2 water at 37 °C. The reaction mixture was shaken at 800 rpm for 420 min, and the adsorption kinetics of the surface interaction were evaluated by determining the residual OTA concentration with UV/Vis spectrophotometry at 30 min intervals. Elovich, pseudo-first-order, and pseudo-second-order models were used to analyze the adsorption dynamics of the OTA/clay interaction [29,57,58].

### 5.6. Data Calculations and Curve Fitting

The nonlinear pseudo-first-order rate equation’s expression is shown as follows:

q_t_ = q_e_ × [1 − exp (−K_1_ × t)] (1)

where *q_e_* and *q_t_* denote the amounts in mg/kg of OTA adsorbed onto the binding surfaces of various sorbents at equilibrium and time *t* (min). The rate constant of the first order is *K*_1_ (min^−1^).

The nonlinear pseudo-second-order rate equation’s expression is shown as follows:

(2) $$q_{t}=\frac{K_{2}\times qe^{2}\times t}{1+qe\times K_{2}\times t}$$

where *q_e_* and *q_t_* denote the amounts in mg/kg of OTA adsorbed onto the binding surfaces of various sorbents at equilibrium and time *t* (min). The rate constant of the second order is *K*_2_ (mg kg^−1^ min^−1^).

The expression for the Elovich equation is as follows:

q_t_ = 1/b ln ab + 1/b ln t (3)

This study involves calculating the initial adsorption rate represented by the symbol a (in mg/kg min) and the parameter b, which are related to the amount of surface covered and the activation energy involved in chemisorption (in mg kg^−1^). The kinetic parameters of nonlinear models were determined by a trial-and-error method of computational operations. By minimizing the squared deviation and the coefficient of determination between the observed experimental data and the expected values during computer simulations, the parameters were determined.

The OTA concentration was calculated by comparing the concentration (mol kg^−1^) of the test and control groups from isotherm plots. To investigate the adsorption data and determine certain parameters, Table-curve 2D V. 5.01 (Systat Software, Inc., Palo Alto, CA, USA) and the R programming language were used. The adsorption data were computed using the R code, and their conformance to established and well-known models was verified using maximum-likelihood estimation. Using the information matrix approach, standard errors and confidence intervals were computed [59,60]. The Langmuir model was used to display adsorption isotherms based on the mean of the observed data points and the 95% confidence intervals from triplicate investigations. The adsorption of a monolayer on a surface with homogenous adsorption energies and a finite number of identical sites is depicted by the Langmuir model. A homogenous surface with the least amount of interaction between the adsorbed species is indicated by adsorption sites with the same energy level in a perfect Langmuir scenario.

(4) $$Langmuir\;model\;q=Qmax\;\frac{K_{d}C_{w}}{1+K_{d}C_{w}}$$

where *C*_w_ = equilibrium concentration of OTA (mol L^−1^), *K*_d_ = Langmuir distribution constant, *Q*_max_ = maximum binding capacity (mol kg^−1^), and *q* = the amount of OTA adsorbed (mol kg^−1^).

To determine *Ke°* from *K_d_*, the following equation was used:

(5) $$Ke{}^{\circ}=\frac{K_{d}{\left[adsorbate\right]}^{o}}{\gamma }$$

where *Ke°* is the thermodynamic equilibrium constant, [*adsorbate*]° is the standard concentration of the adsorbate = 1 mol L^−1^, and *γ* is the coefficient of activity.

The enthalpy (Δ*H*) and free energy (Δ*G°*) were determined by using the Gibbs free energy equation together with the adsorption parameters and van ’t Hoff equation as given below:

ΔG = ΔG° + RTInKe°(6)

(7) $$\Delta H=\frac{-RIn(\frac{K_{d2}}{K_{d1}})}{\left(\frac{1}{T_{2}}\right)-(\frac{1}{T_{1}})}$$

where *T* (absolute temperature) = 273 + *t* (°C) and *R* (gas constant) = 8.314 J mol^−1^ K^−1^. When Δ*G°* is positive, the adsorption process is not favored and could not be significant (Ke° < 1). However, if the value is negative, it is an indication that adsorption process is enhanced thermodynamically and is proceeding forward (Ke° > 1). Δ*G* will be zero for an adsorption system in equilibrium.

### 5.7. Hydra Vulgaris In Vivo Assay

The *Hydra vulgaris* utilized in this research were sourced from Environmental Canada in Montreal and were maintained at a consistent temperature of 18 °C. Using a comprehensive scoring system ranging from 0 to 10, the morphological features of hydra were used as biomarkers for the toxicity of different chemicals. When the hydra is healthy and has lengthy tentacles, this method gives it a score of 10, and when it appears to be dead and disintegrated, it gives it a score of 0. In this study, hydra were subjected to different OTA concentrations ranging from 0 to 15 ppm (g/mL) at 1, 2.5, 5, 7.5, 10, and 15 ppm (g/mL) in order to investigate the toxicological profile of OTA. The minimum effective dose that led to the complete mortality of hydra within 92 h was determined and used for detoxification assessment with sorbent inclusions at 0.2% (weight/volume) to 0.5% (weight/volume). In these studies, OTA was introduced to the hydra as a mixture of OTA and sorbent. Each experimental group consisted of three hydra colonies at 18 °C in 4 mL of testing media. Average scores were recorded at exact intervals (0, 4, 20, 28, 44, 68, and 92 h) to evaluate the levels of OTA toxicity and protection provided by the inclusion of each sorbent.

### 5.8. Caenorhabditis Elegans In Vivo Assay

The toxicity of OTA and protective effect of both the parent clays and engineered sorbents were examined in this study using nematodes as an in vivo model. *Caenorhabditis elegans* are well-established and sensitive organisms for mycotoxin toxicity testing [52]. The *E. coli* strains NA22 and OP50-1, as well as the wildtype (Bristol N2) *C. elegans*, were obtained from the Caenorhabditis Genetics Centre (CGC, University of Minnesota). The protocol reported by Wang et al. [61] was used for the synchronization of the worms. Briefly, the nematodes were cultured on 8P media containing 8 × 10^8^ cells/mL of *E. coli* NA22 and were kept at a temperature of 20 °C. Bleaching and washing were used to produce age-synchronized L1 nematodes. For eighteen hours at 20 °C, egg culture solutions were shaken at 2.5 rpm on a rocking platform (VWR, Radnor, PA, USA) in the dark.

Following the incubation time, OTA dosimetry and toxic effect on *Caenorhabditis elegans* were investigated. Briefly, the worms were counted and then placed in an Eppendorf tube to be exposed to various concentrations of OTA ranging from 0 ppm to 100 ppm at 12.5, 25, 50, 75, and 100 ppm. An appropriate quantity of K media and *E. coli* OP50-1 was added into the tube at 20 °C and shaken at 2.5 rpm on a rocking platform in the dark for a duration of 24 and 48 h, respectively. By counting the number of worms drawn by a coated 10 µL pipette, the survival rate was extrapolated. Using the nose contact technique, the locomotion of ten nematodes per group was assessed under an Olympus SZ61 zoom stereomicroscope (Olympus, Waltham, MA, USA). The separated nematodes were then placed on a fresh agar plate and given a 24/48 h period to develop at 20 °C. After the nematodes were paralyzed with 25 mM sodium azide, the body length of each individual nematode was measured using the CellSens Entry (standard version 3) program (Olympus, Waltham MA, USA). In order to adjust to 100%, the relative body lengths were computed as a percentage of the medium control group. Detoxification efficacy of the parent clays and engineered sorbents was assessed using the same protocol as described in the toxicity examination with the inclusion of 0.2% and 0.5% of sorbents. Based on the toxicity results, 25 ppm of OTA was selected for the detoxification studies.

### 5.9. In Silico Studies of OTA onto the Binding Surfaces of Chlorophyll-Amended Clay

Computational studies were used to investigate OTA in complex with parent clay (CM) and chlorophyll-amended clay (CMCH) prompted by in vitro studies. Particularly, chlorophyll-A was considered both in its natural form at neutral conditions (CH) and as pheophytin-A (PHO); see Figure S1. Certain conditions such as high temperature, pH, and enzymatic reactions could cause conversion of CH to PHO and both could co-exist [62]. Thus, this in silico investigation included both chlorophyll-amended (CMCH) and pheophytin-amended (CMPHO) clay at pH 3 and 7. CM clay is available from Interface-FF [63] at both acidic (pH 3) and neutral (pH 7) conditions. As for CMCH and CMPHO, initial simulations, as described below, were used to construct both amended clays. The two amended clays were investigated independently, and in this way, the computational studies advantageously enabled us to understand similarities and any potential differences stemming from the two possible states that the molecule may exist.

In acidic conditions, clay was considered and thus investigated in its acidic state while OTA molecules were neutral (uncharged) in the system. In neutral conditions, clay was considered and thus investigated in its neutral state while OTA molecules were considered as either monoanionic or dianionic [64]. The structures of the three states of OTA are shown in Figure S2. OTA binding to parent clay without amendments in both acidic and neutral conditions served as a reference point (i.e., control). The described systems and corresponding simulated conditions are shown in Figure S3.

Using our previous studies as a basis [28,56,65], acidic clay was initially constructed using CHARMM-GUI [63,66,67,68,69] according to the provided INTERFACE FF [63] model: a layer of CM with the chemical composition (Si_4_)^IV^(Al_1.67_Mg_0.33_)^VI^O_10_(OH)_2_, dimension 50 × 50 Å^2^, Miller indices 001, and a ratio of defect of 0.33333. For neutral clay, particular hydrogens and hydroxyl groups from the clay edges of the acidic model were manually removed, according to INTERFACE FF [63], and a separation distance of 21 Å was used as in our previous studies [28,54,65]. Na^+^ provided by default by CHARMM-GUI [63,66,67,68,69] were manually deleted and within the explicit solvent simulations, 20 Ca^2+^ (one for every two Na^+^) were introduced randomly as part of the simulation setup, taking into consideration the clay layer dimensions used. In the simulations of all systems under investigation, Ca^2+^ ions were unconstrained and capable of freely moving away from clay layers. Additional Ca^2+^ ions were added, when necessary, to neutralize the constructed systems, based on the number of molecules added and their corresponding charge in each case. The INTERFACE FF [63] force field was used for CM parameterization in all computational studies, and parameters for ions were obtained [70]. Input files to set up and simulate the aforementioned systems were used in accordance with previous studies [28,56,65]; they were initially generated using CHARMM-GUI [63,66,67,68,69], and were modified, as described below, to study CM, CMCH, and CMPHO.

Similarly to our previous published studies for chlorophyll-A [28,56,65], the model structure for chlorophyll was extracted from Protein Data Bank (PDB), corresponding to molecule entry CLA. The topology and parameter files for chlorophyll were initially obtained using a combination of parameters from CGenFF [71], and charges for the chlorin ring were obtained from Guerra et al., [72] while ensuring the molecule’s net charge was kept to zero [54]. Throughout the simulations, magnesium was constrained with reference to the four nitrogen neighboring atoms at a distance equal to 2.107 Å, corresponding to the average distance of the model PDB structure of the molecule. This was performed using MMFP harmonic constraints in CHARMM, and distance constraints with harmonic bond force in OpenMM. The pheophytin model structure was extracted from PDB, corresponding to molecule entry PHO, and the topology and parameter files were obtained using CGenFF. The structure for uncharged OTA was obtained from PDB, corresponding to molecule entry 97U. Subsequently, MolGpKa [73] was used to predict the pK_a_ values of each ionizable group of OTA in order to remove the proper hydrogens for each of the monoanionic and dianionic states of OTA. CHARMM-GUI Ligand Reader and Modeler was used to ionize OTA and create the two new structures for the two anionic states [63,66,67,68,69]. Subsequently, CGenFF [71] was utilized to parametrize the two different states of OTA. For uncharged OTA, the topology and parameter files were obtained using CGenFF [71].

To amend CH and PHO to CM, clay was initially centered in a cubic (90 Å) periodic boundary condition box and solvated by explicit water molecules (TIP3P model) [69] in CHARMM [68]. Twelve amendment molecules with different configurations, produced through short elevated-temperature MD simulations of each molecule, were randomly placed and oriented in the box. Water molecules in close proximity to the clay or the amending molecules were removed prior to conducting simulations, and a combination of SD and ABNR energy minimization steps were conducted in CHARMM [68] prior to simulations. Triplicate MD simulation runs were performed as follows. Equilibration runs (NVT) were performed for 200 ps in CHARMM at 300 K, during which harmonic constraints were introduced to aluminum and magnesium atoms of the clay with an input force constant of 1 kcal/mol Å^2^. Subsequently, production runs (NPT) were performed in OpenMM [74] for 60 ns at 300 K and 1 atm (isotropic barostat), during which magnesium and aluminum atoms of clay were constrained with a force constant of 400 kJ/mol nm^2^. We used visual inspection to select the optimally amended CMCH and CMPHO structures from the last simulation snapshots of triplicate runs per amended clays (Figure S4). These were used as initial structures in the simulations described below to investigate OTA binding, upon recentering chlorophyll molecules and adjusting clay layers’ position.

To investigate OTA molecules binding to amended clays (i.e., CMCH and CMPHO), two and four systems were investigated at acidic and neutral conditions, in complex with an acidic and neutral amended clay, respectively (Figure S3). The two systems in acidic conditions correspond to uncharged OTA in complex with acidic CMCH and acidic CMPHO. The four systems in neutral conditions correspond to monoanionic and dianionic OTA in complex with neutral CMCH and neutral CMPHO (Figure S3). For each system, we performed triplicate runs as follows. The initial structures obtained above corresponding to acidic or neutral amended clay (CMCH or CMPHO) in a cubic (90 Å) periodic boundary conditions box were solvated by explicit water molecules (TIP3P model) [75] in CHARMM [68]. Sixteen OTA molecules (in the corresponding state) with different configurations, produced through short elevated-temperature MD simulations of each molecule, were randomly placed and oriented in the box. Water molecules in close proximity to the clay, the amending, or OTA molecules were removed prior to conducting simulations, and a combination of SD and ABNR energy minimization steps were conducted in CHARMM [68] prior to simulations. Equilibration runs (NVT) were performed for 200 ps in OpenMM [74] at 300 K, during which harmonic constraints were introduced to aluminum and magnesium atoms of the clay with an input force constant of 400 kJ/mol nm^2^. Subsequently, production runs (NPT) were performed in OpenMM [74] for 100 ns at 300 K and 1 atm (isotropic barostat), during which magnesium and aluminum atoms of clay were constrained with a force constant of 400 kJ/mol nm^2^. In each case, triplicates MD simulations started from the same initial structure as a result of the amending process. Additionally, to investigate OTA binding to CM, which we refer to as control runs, we used an analogous setup to the one described above, i.e., 16 OTA molecules were investigated in complex with CM (in the absence of any amendments) using triplicate 100 ns runs. One and two systems were investigated under acidic and neutral conditions, in complex with an acidic and neutral clay, respectively. The system in acidic conditions corresponds to uncharged OTA in complex with acidic CM, while the two systems in neutral conditions correspond to monoanionic and dianionic OTA in complex with neutral CM (Figure S3).

Upon completion of the simulations, in-house FORTRAN programs were utilized to investigate interactions of OTA with CM, CMCH, and CMPHO. Simulation trajectories were analyzed with snapshots extracted every 1 ns; thereby, 100 snapshots were analyzed per simulation. We determined the average binding percentage probability of OTA in each applicable protonation state to each one of the parent and amended clays. The binding percentage probability was calculated by counting the total number of instances that an OTA molecule was in contact with the parent and amended clays, and it was normalized by the total number of OTA molecules (16) in the simulations and the total number of snapshots analyzed (100). Then, the average binding percentage probability was calculated by averaging the binding percentage probabilities over the triplicate simulations per investigated system. Two entities were considered to be in contact, and thus interact with each other, when the distance between any pair of their atoms (including hydrogens) was ≤3.5 Å. Overall, our programs tracked interactions between (a) OTA and CH or PHO molecules; (b) OTA and CM, which are referred to as “direct” interactions; and (c) OTA molecules. Additionally, the programs tracked potential calcium-mediated interactions between (d) OTA and CH or PHO molecules. Interactions associated with (a) were further decomposed into (i) direct-assisted interactions, which are interactions of OTA with CM and amendment molecules (bound to the clay) simultaneously; (ii) indirect-assisted interactions, which are interactions of OTA only with amendment molecules, which are either bound directly to clay or they are part of a cluster of amendment molecules indirectly bound to clay. A recursive function was developed to identify clusters of amendment molecules and clusters of OTA molecules. For example, if an OTA molecule was participating in a direct-assisted or in an indirect-assisted interaction and one or multiple other OTA molecules was/were interacting with that OTA molecule, then they were also considered as if they were participating in an indirect-assisted interaction. To avoid double or multiple counting in cases where an OTA molecule was found to interact in more than one way with CMCH or CMPHO, direct (b) or direct-assisted (ai) interactions were prioritized over indirect-assisted interactions (aii). To obtain additional insight into the chemical groups contributing to interactions in (a), we additionally decomposed OTA molecules into five groups: two hydrophobic groups, two hydrophilic groups, and the chlorine atom. This chemical decomposition into chemical groups is shown in Figure S2. The decomposition enabled better understanding of the mechanisms behind the interactions of OTA with parent clay (CM), CH, and PHO. In this case, chemical groups of OTA and the amendments were considered to be in contact, and thus interact with each other, when the distance between any pair of the atoms within the chemical groups (including hydrogens) was ≤3.5 Å. Notably, while this can be a rather strict criterion for polar interactions involving calcium with OTA, the same criterion was used for all interactions for consistency. In (d), a calcium-mediated interaction between CH/PHO and OTA was considered if the distance between a particular calcium with any atom of CH/PHO and OTA was below the cutoff. Snapshots of simulations were obtained and visualized using VMD [76].

### 5.10. Statistical Analysis

In this investigation, each trial including the blank and negative controls was conducted independently at least three times. One-way ANOVA assessed the statistical significance of the data, followed by a post hoc Tukey test. Key parameters such as *K_d_* and *Q_max_* derived from adsorption and thermodynamic isotherms, along with scores from toxicity and detoxification assessments of hydra, were analyzed for standard deviation and *p*-values. The Bonferroni analysis was applied for multiple comparisons, and the results were considered significant at *p* < 0.05.

## Figures and Tables

**Table 1:** Physicochemical properties of sorbents.

S/N	Clay Properties	CMCH	CMCHin	SMCH	SMCHin	CM	SM
1	Appearance	Green powder	Green powder	Green powder	Green powder	Off-white to greyish green	Off-white
2	Hydrophobicity	2.15	0.72	1.61	0.79	0.45	0.27
3	pH	6.99	8.57	6.98	9.02	6.93	6.87
4	pHpzc	9.70	9.80	7.80	12.10	8.20	8.90
5	COLE	1.20	1.50	1.40	6.00	1.00	7.00
6	Moisture content (%)	2.69	2.96	2.32	2.98	1.08	1.43
7	Density (kg/m^3^)	536	463	683	592	908	848

CM: calcium montmorillonite; CMCH: chlorophyll-amended calcium montmorillonite; CMCHin: chlorophyllin-amended calcium montmorillonite; SM: sodium montmorillonite; SMCH: chlorophyll-amended sodium montmorillonite; SMCHin: chlorophyllin-amended sodium montmorillonite.

**Table 2:** Parameters and correlation coefficients of adsorption isotherms.

Sorbents	Adsorption Parameters				Desorption Parameters		
**Langmuir**							
	Q_max_	K_d_	∆G	r^2^	∆K_d_ (%)	∆Q_max_ (%)	Binding percentage (%)
CMCH	1.33	9.02 × 10^5^	−32.42	0.74	100	78	99
CMCHin	1.15	8.40 × 10^6^	−32.98	0.96	16	100	90
SMCH	2.13	1.15 × 10^7^	−34.26	0.99	100	82	93
SMCHin	0.73	6.47 × 10^7^	−29.74	0.53	16	78	73
**Freundlich**							
	1/n	K_f_	∆G	r^2^	∆K_f_ (%)	∆1/n (%)	Binding percentage (%)
SM	0.96	2.43 × 10^5^	−31.70	0.63	0	NA	0
CM	0.62	6.18 × 10^7^	−27.96	0.67	NA	59	89

K_f_: Freundlich distribution constant; 1/n: degree of heterogenicity; r^2^: correlation coefficients; ∆G: Gibbs free energy (kJ/mol); K_d_: binding affinity; Q_max_: binding capacity (mol/kg). CM: calcium montmorillonite; CMCH: chlorophyll-amended calcium montmorillonite; CMCHin: chlorophyllin-amended calcium montmorillonite; SM: sodium montmorillonite; SMCH: chlorophyll-amended sodium montmorillonite; SMCHin: chlorophyllin-amended sodium montmorillonite.

**Table 3:** Adsorption kinetic parameters for OTA on the sorbents.

Pseudo-Second-Order Parameters	qe (exp mg·kg^−1^)	qe (cal mg·kg^−1^)	K_2_	r^2^
CM	0.88	0.82	5.21 × 10^−2^	0.85
CMCH	0.80	0.76	3.24 × 10^−2^	0.92
CMCHin	0.94	0.80	2.34 × 10^−2^	0.73
SMCH	0.93	0.75	3.98 × 10^−2^	0.87

r^2^: correlation coefficients; qe (exp): binding at equilibrium in the experiment; qe (cal): binding at equilibrium calculated by the pseudo-second-order model; K_2_: rate constant of the second order; CM: calcium montmorillonite; CMCH: chlorophyll-amended calcium montmorillonite; CMCHin: chlorophyllin-amended calcium montmorillonite; SMCH: chlorophyll-amended sodium montmorillonite.

## Data Availability

The original contributions presented in this study are included in the article/supplementary material. Further inquiries can be directed to the corresponding author(s).

## Supplementary Materials

The following supporting information can be downloaded at: https://www.mdpi.com/article/10.3390/toxins16110479/s1, Figure S1: Structure of (A) Chlorophyll A and (B) Pheophytin A; Figure S2: The top parts of panels A–C depict the molecular structures of uncharged, monoanionic, and dianionic OTA, respectively. The corresponding bottom parts of panels A–C present the decomposition of the molecule into different chemical groups; Figure S3: This scheme depicts the workflow of the simulated systems. Panel A shows the amending process of acidic clay with chlorophyll molecules (CMCH), while panel B shows the amending process of acidic clay with pheophytin molecules (CMPHO); Figure S4: (A) Chlorophyll-amended clay (CMCH); (B) pheophytin-amended clay (CMPHO); Figure S5: Snapshots extracted from simulations of OTA in complex with CM, referred to as control simulations. Panels A, B, and C correspond to acidic conditions, as well as neutral conditions simulating monoanionic and dianionic OTA, respectively.
