# Supplementary material for: Chlorophyll-Amended Organoclays for the Detoxification of Ochratoxin A
Source: Toxins (Basel). 2024 Nov 6;16(11):479. doi: 10.3390/toxins16110479 (PMC11598794; doi:10.3390/toxins16110479)
Supplement: Supplementary file 1 [file toxins-16-00479-s001.zip › toxins-3255256-supplementary.pdf]

Supplementary Materials: Chlorophyll-Amended Organoclays for the Detoxification of Ochratoxin A

Johnson O. Oladele, Meichen Wang, Xenophon Xenophontos, Kendall Lilly, Phanourios Tamamis, Timothy D. Phillips

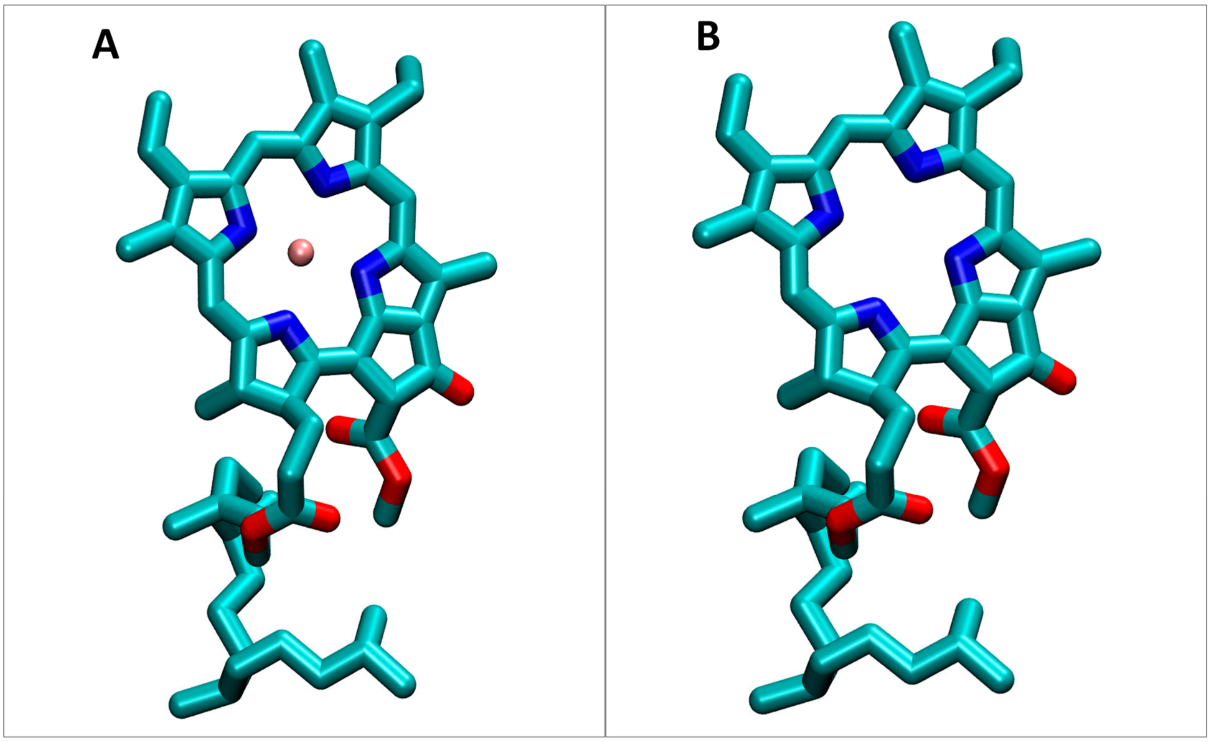

Figure S1. Structure of (A) Chlorophyll A and (B) Pheophytin A. The sphere shown at the center of chlorophyll's chlorin ring corresponds to magnesium, and the rest of the covalently bonded atoms are shown in licorice representation, colored by atom type. Hydrogens are omitted for clarity.

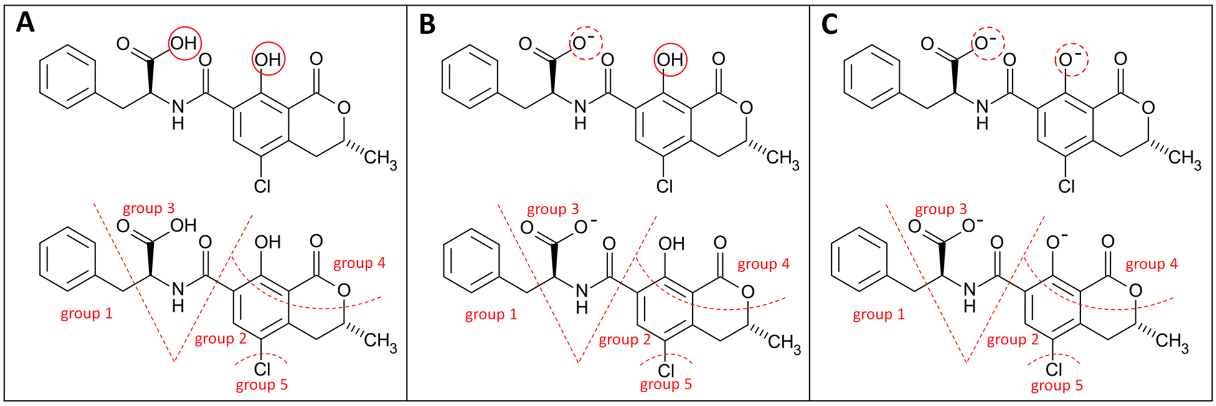

Figure S2. The top parts of panels A–C depict the molecular structures of uncharged, monoanionic, and dianionic OTA, respectively. The corresponding bottom parts of panels A–C present the decomposition of the molecule into different chemical groups.

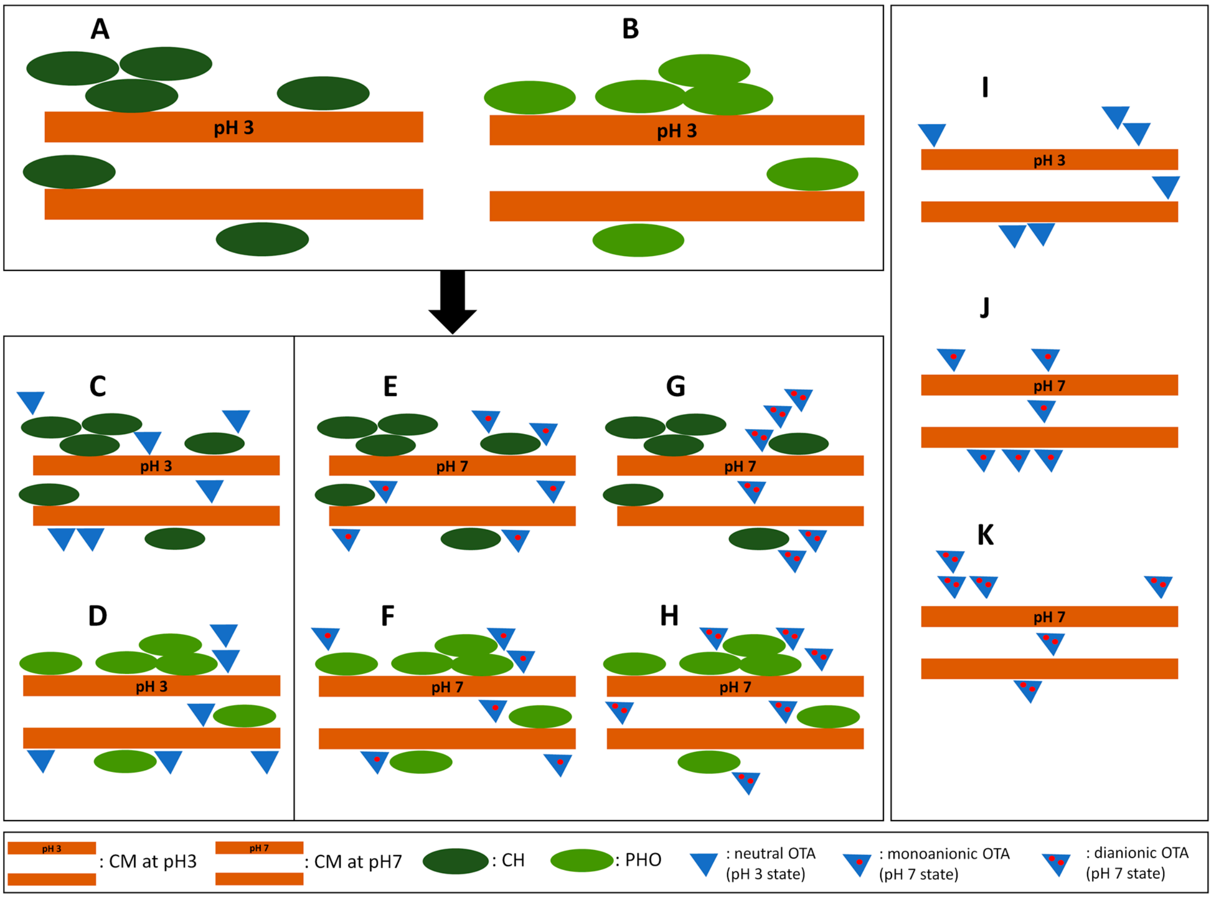

Figure S3. This scheme depicts the workflow of the simulated systems. Panel A shows the amending process of acidic clay with chlorophyll molecules (CMCH), while panel B shows the amending process of acidic clay with pheophytin molecules (CMPHO). Panels C and D show simulated systems investigating interactions between OTA and CMCH and CMPHO, respectively, in acidic conditions; for these simulations, acidic clays was were used, and OTA was set in its neutral (uncharged) state. Panels E-–H show simulated systems investigating interactions between OTA and CMCH and CMPHO in neutral conditions; for these simulations, neutral clay was used instead. Panels E and F show simulations where monoanionic OTA was tested with CMCH and CMPHO, respectively, while Panels G and H show the simulations where dianionic OTA was tested with CMCH and CMPHO, respectively. Panels I-–K show control simulated systems investigating interactions between OTA and CM. Panel I shows simulated systems investigating interactions between uncharged OTA with an acidic CM, while panels J and K show simulated systems investigating the interaction of monoanionic and dianionic OTA, respectively, with neutral CM.

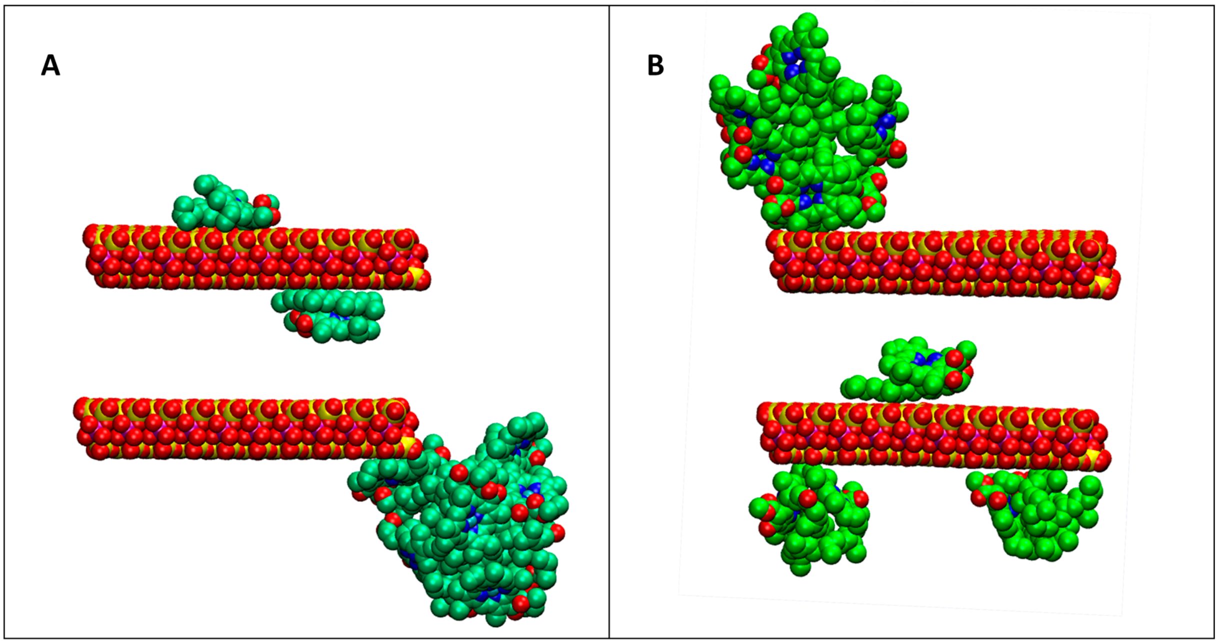

Figure S4. (A) Chlorophyll-amended clay (CMCH); (B) pheophytin-amended clay (CMPHO). Molecules are represented in vdW representation, with atoms colored by type, with the exception of carbon atoms, which are shown in green and light green for CH and PHO, respectively. Hydrogen atoms are omitted for clarity.

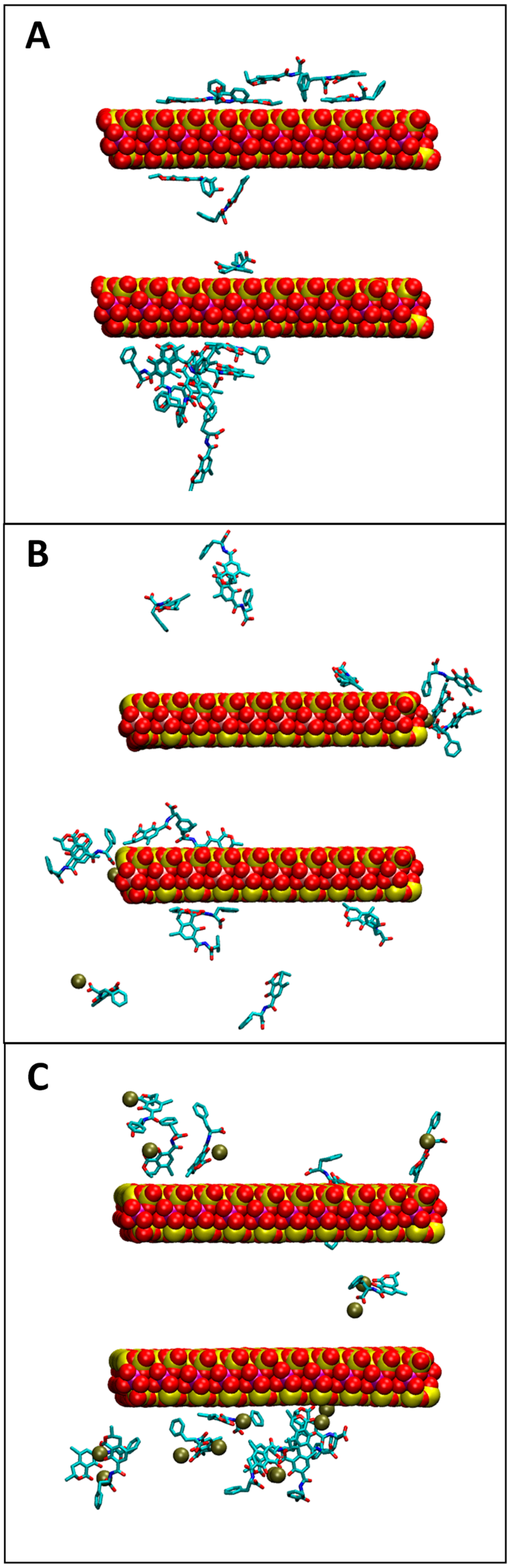
A
B
C

Figure S5. Snapshots extracted from simulations of OTA in complex with CM, referred to as control simulations. Panels A, B, and C correspond to acidic conditions, as well as neutral conditions simulating monoanionic and dianionic OTA, respectively. CM and calcium are shown in vdW representation, while OTA is shown in licorice representation. Atoms are colored by atom type, apart from calcium ions, which are colored in tan. Calcium ions that are greater in distance than 3.5 Å from all OTA molecules are omitted. Hydrogen atoms are also omitted for clarity.
